# Voltage‐gated proton channels in polyneopteran insects

**DOI:** 10.1002/2211-5463.13361

**Published:** 2022-01-19

**Authors:** Gustavo Chaves, Christian Derst, Christophe Jardin, Arne Franzen, Boris Musset

**Affiliations:** ^1^ Center of Physiology, Pathophysiology and Biophysics Paracelsus Medical University Nuremberg Germany; ^2^ Institute of Biological Information Processing (IBI‐1), Molekular‐ und Zellphysiologie Forschungszentrum Jülich Germany; ^3^ Center of Physiology, Pathophysiology and Biophysics Paracelsus Medical University Salzburg Austria

**Keywords:** EtH_V_1, glutamate, H_V_1, polyneopteran insects, selectivity, voltage‐gated proton channels

## Abstract

Voltage‐gated proton channels (H_V_1) are expressed in eukaryotes, including basal hexapods and polyneopteran insects. However, currently, there is little known about H_V_1 channels in insects. A characteristic aspartate (Asp) that functions as the proton selectivity filter (SF) and the RxWRxxR voltage‐sensor motif are conserved structural elements in H_V_1 channels. By analysing Transcriptome Shotgun Assembly (TSA) databases, we found 33 polyneopteran species meeting these structural requirements. Unexpectedly, an unusual natural variation Asp to glutamate (Glu) at SF was found in Phasmatodea and Mantophasmatodea. Additionally, we analysed the expression and function of H_V_1 in the phasmatodean stick insect *Extatosoma tiaratum* (Et). EtH_V_1 is strongly expressed in nervous tissue and shows pronounced inward proton conduction. This is the first study of a natural occurring Glu within the SF of a functional H_V_1 and might be instrumental in uncovering the physiological function of H_V_1 in insects.

## Graphical abstract

We report the discovery of voltage‐gated proton channels (H_V_1) in polyneopteran insects. We found a novel, naturally occurring glutamate in the selectivity filter of H_V_1. We further characterized the H_V_1 of the phasmatodean *Extatosoma tiaratum*, EtH_V_1. This study provides the first step in the discovery of the physiological function of H_V_1 channels in insects and its relationship with their evolution.

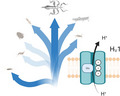

**Abbreviations**
Ala: alanine
Arg: arginine
Asp: aspartate
BIS‐TRIS: Bis‐(2‐hydroxyethyl)imino‐tris‐(hydroxymethyl)‐methane
EGTA: Ethylene glycol‐bis(2‐aminoethylether)‐N,N,N′,N′‐tetraacetic acid
*E*_H_
: Nernst potential for protons
EtH_V_1: *Extatosoma tiaratum* voltage‐gated proton channel
*g*_H_
: proton conductance
GHK: Goldman–Hodgkin–Katz
Glu: glutamate
His: histidine
H_V_1: voltage‐gated proton channels
MES: 2‐(N‐morpholino)ethanesulfonic acid
P_CO2_
: partial CO_2_ pressure
pH_i_
: internal pH
pH_o_
: external pH
PIPES: piperazine‐N,N′‐bis(2‐ethanesulfonic acid)
S1, S2, S3, S4: transmembrane alpha helices 1, 2, 3 and 4
SF: selectivity filter
TSA: transcriptome shotgun assembly
*V*_rev_
: reversal potential
*V*_thres_
: threshold potential
τ*_act_*
: activation time constant

Voltage‐gated proton channels are found in most eukaryote kingdoms, from coccolithiophores [1] to dinoflagellates [2], chordata, fungus, plants and mammals [3, 4]. In dinoflagellates (*Lingulodinium polyedrum*), H_V_1 channels trigger light emission [5]. In mammals, H_V_1 channels play a pivotal role in many physiological processes including pH homeostasis, respiratory burst of phagocytes and maturation of sperm [6, 7, 8]. In several breast and colorectal cancers, H_V_1 is significantly upregulated [9, 10]. Much less is known about H_V_1 channels in hexapoda, especially in insects. Recently, a H_V_1 channel of the Zygentoma *Nicoletia phytophila* was characterized and other H_V_1 sequences have been found in phylogenetically more basal hexapodes [11].

In this publication, we focus mainly on H_V_1 expression in Polyneoptera, a major lineage of winged insects evolved ~ 400 millions of years ago [12, 13]. It includes grasshoppers/crickets/locusts (Orthoptera), stoneflies (Plecoptera), earwigs (Dermaptera), cockroaches and termites (Blattodea), mantis (Mantodea), stick and leaf insects (Phasmatodea), gladiators or heelwalkers (Mantophasmatodea), webspinners (Embioptera), ice crawlers (Grylloblattodea) and ground lice (Zoraptera). Besides basal hexapodes, insect H_V_1 homologs are mainly found within this insect lineage [11]. Interestingly, the well‐characterized dipteran genomes of insect model systems and disease carriers such as *Drosophila*, *Aedes* and *Anopheles* do not possess a typical H_V_1 gene [14, 15, 16].

As a member of the voltage‐gated superfamily of ion channels, H_V_1 possesses four transmembrane regions (S1–S4) with a typical voltage‐sensor element in the fourth transmembrane segment (S4) [3, 4]. Compared to other voltage‐gated ion channels, such as potassium, sodium and calcium channels, H_V_1 does not have the last two transmembrane regions (S5–S6) of the typical six transmembrane alpha helices, usually composing the pore region. Instead, a different ion conduction pathway is established for protons, including all four transmembrane regions with a typical negatively charged aspartate in S1 as proton selectivity filter (SF) [17, 18, 19]. Additional site‐directed mutagenesis of this aspartate residue (position 112 in human H_V_1) revealed new aspects of structure–function relationships within H_V_1 channels [2, 11, 17, 20, 21]. To date, H_V_1 channels are considered to be dimeric with the intracellular C‐terminal domain connecting both subunits [22, 23, 24].

The aim of this study was to characterize the H_V_1 channel of the stick insect *Extatosoma tiaratum*. Besides the H_V_1 channel of the Zygentoma *Nicoletia*, this is the second H_V_1 channel from Hexapoda and the first of the class Insecta. Our database analysis provided a detailed picture of the presence and absence of H_V_1 genes in different hexapodan and insect orders. With the analysis of the tissue‐specific expression in *Extatosoma* and the electrophysiological characterization, we hope to blaze a trail for uncovering the physiological function of H_V_1 channels in insects. The presence of an unusual glutamate residue as SF within the S1 domain of *Extatosoma* H_V_1 was analysed and compared to the other hexapodan H_V_1 channel from *Nicoletia*.

## Materials and methods

### Database analysis

The BLAST algorithm was used to analyse insect transcriptome shotgun assembly (TSA) and genomic databases at NCBI. The recently described sequence of the Zygentoma *Nicoletia phytophila* (KT780722 [11, 25]) was used as query sequence. Only sequences harbouring the typical RxWRxxR motif in the S4 segment were used for further analysis.

### Tissue expression analysis

Two *Extatosoma* animals were manually dissected and five tissues (ganglia/nervous system, leg muscle, eyes, antenna and digestive system) were isolated. Tissues from both animals were combined. Total RNA of the five tissue samples was isolated using the RNeasy MiniKit (Qiagen, Hilden, Germany) and reverse‐transcribed using Sensiscript reverse transcriptase (Qiagen) and random hexanucleotides (Roche, Basel, Switzerland). The resulting cDNA was used as a template for a PCR analysis using *Extatosoma tiaratum* voltage‐gated proton channel (EtH_V_1)‐specific primers (forward: 5′‐ATGGACAGCTGGAATGTGGA‐3′, reverse: 5′‐CCATGTATGTACTGCGCTGC‐3′), as a positive control the ubiquitous expression of histone H3 was analysed (forward: 5′‐CAAGTCGACTGGAGGCAAAG‐3′, reverse: 5′‐TGGGCATGATGGTGACTCTT‐3′). A standard PCR protocol was conducted using Advantage Taq polymerase mixture (TaKaRa) and 55 °C annealing temperature. The resulting PCR products were analysed on a 2% agarose gel and inspected for 363‐bp bands (EtH_V_1) and 345‐bp bands (histone H3).

### Heterologous expression

*Extatosoma tiaratum* EtH_V_1 gene was synthesized commercially (Eurofins/Genomics, Ebersberg, Germany). The synthesized DNA including a 5′ *Bam*HI and 3′ *Eco*RI restriction site was cloned into a pEX‐A2 plasmid. The gene was later subcloned into a pQBI25‐fC3 or pcDNA3.1, using 5′*Bam*HI and 3′ *Eco*RI restriction sites and GFP fused to N‐terminal as previously described [2, 11, 17, 25]. tSA201 cells (human kidney cell line) were grown to 85% confluency in 35 mm culture dishes. Cells were transfected with 1.0 μg plasmid DNA using polyethylenimine (Sigma, St. Louis, MO, USA). After 12 h at 37 °C in 5% CO_2_, cells were trypsinized and replated onto glass coverslips at low density for patch clamp recording the same day and the next day. Green cells were selected under fluorescence for recording. As in [11, 25], whole cell patch clamp showed no other voltage‐ or time‐dependent conductance under our recording conditions. The level of expression of EtH_V_1 was sufficiently high so that potential contamination by native H_V_1 currents was negligible.

### Electrophysiology

Patch‐clamp recordings were done as described in [11, 25]: A patch‐clamp amplifier EPC 10 (HEKA, Lambrecht, Germany) was used. Recordings were stored on hard discs and analysed with Origin (Origin 2017, Northampton, MA, USA). Patch pipettes were made from borosilicate capillaries GC 150TF‐10 (Harvard Apparatus, Holliston, MA, USA) and pulled using Flaming Brown automatic pipette puller P‐1000 (Sutter Instruments, Novato, CA, USA). Pipettes were heat polished to a tip resistance ranging typically from 5 to 9 MΩ with pipette solutions used. Electrical contact with the pipette solution was achieved by a chlorinated silver wire and connected to the bath with an agar bridge made with Ringer’s solution. Seals were formed with Ringer’s solution (in mm 160 NaCl, 4.5 KCl, 2 CaCl_2_, 1 MgCl_2_, 5 Hepes, pH 7.4) in the bath, and the potential zeroed after the pipette was placed above the cell. Whole‐cell and inside‐out solutions (pipette and bath) included 100 mm buffer close to its p*K*_a_ with tetramethylammonium (TMA^+^) and methanesulfonate (CH_3_SO_3_^−^) as the main ions, 1 mm EGTA, and 1–2 mm Mg^2+^ with an osmolarity of 300 mOsm·kg^−1^. Buffers were 2‐(N‐morpholino)ethanesulfonic acid (MES) at pH 5.5 and pH 6.0, Bis‐(2‐hydroxyethyl)imino‐tris‐(hydroxymethyl)‐methane (BIS‐TRIS) at pH 6.5 and PIPES at pH 7.0. Resistance of the seals was usually > 3 GΩ. Currents are shown without correction for leak or liquid junction potentials. Data were collected between 19 °C and 23 °C. Currents were fitted to a rising exponential to obtain the activation time constant (τ*_act_*). The maximal proton conductance (*g*_H, max_) was calculated from the steady‐state current (the fitted current extrapolated to infinite time) using reversal potentials (*V*_rev_) measured in each solution in each cell. In these fits, the initial delay was ignored and the remaining current usually fitted a single exponential well. The threshold potential, *V*_thres_, was determined from families of pulses as the potential where the first tail current was observed once the membrane was repolarized. The reversal potential was measured by two methods. When *V*_thres_ was negative to *V*_rev_, it could be readily determined by the zero current. When *V*_thres_ was positive to *V*_rev_, then *V*_rev_ was determined with the tail current method. Voltage dependence of activation was obtained by linear fittings of the activation kinetics plots at the region of the curve where τ*_act_* becomes faster with depolarization. Selectivity for protons was determined by comparison of measured reversal potentials to the Nernst potential for protons (*E*_H_) at the experimental ∆pH (pH_o_ − pH_i_). The pH dependence of gating was evaluated in a pH range from 5.5 to 7.0 by linear regression of data from *V*_thres_ against *V*_rev_ graphs, in a potential range from −70 mV to +70 mV. Overexpression of the channels in small cells resulted in large proton currents which removed enough protons from the cell to change pH_i_ considerably. Proton channel gating kinetics depend strongly on pH; therefore, proton depletion is a significant source of error. To minimize this problem, families with different pulse lengths were applied. Longer pulses were used to determine pulses close to *V*_thres_ where τ*_act_* is slow, while shorter pulses were used at more positive voltages. Zinc inhibition assays were tested extracellular and performed at 0, 10 and 100 μm ZnCl_2_. EGTA was omitted from zinc‐containing solutions. Families of pulses of different lengths were collected in each zinc condition and exchanges of external solutions recorded during test‐pulse protocols. The data are shown without corrections for buffer binding.

### Structural model

A structural model of the transmembrane domain was constructed via homology modelling using Modeller [26, 27] and the crystallographic structure of *Ci*‐VSD in an open state as template (PDB: 4G7V [28]). The amino acid sequences (residues G23 to S158 for *Ci*‐VSD and residues G31 to V169 for *Extatosoma*) were aligned with MUSCLE [29]. A few inaccuracies in the alignment were corrected manually. The two sequences have 18% identity and 40% similarity. 100 models were generated. The best model according to the Modeller objective function was refined with 3DRefine [30]. Five solutions were generated and ranked according to the 3Drefine and RWPlus scores. The solution with the best ranking was conserved.

## Results

### Polyneopteran TSA‐database analysis

Using the typical proton channel signature motif of the voltage sensor in S4 (RxWRxxR), we identified 33 putative polyneopteran H_V_1 channels: nineteen in stick insects (Phasmatodea), eight in locusts/crickets (Orthoptera), three in webspinners (Embioptera), two in gladiators (Mantophasmatodea) and one in stoneflies (Plecoptera). A complete list of all identified channels with the respective GenBank Acc. No. can be found in Table S1. All amino acid sequences compiled from TSA files are shown in Fig. S1. No H_V_1 sequence homolog was found in cockroach TSA databases and in the species‐poor polyneopteran groups of ice crawlers and ground lice. The only sequences found initially in mantis (*Metallyticus splendidus*, GATB01324360, see Table S1) and earwigs (*Forficula auricularia*, GAYQ01077212) were subsequently removed from 1KITE datasets as they most likely represent a fungal contamination of the animal sample ([31], B. Misof personal communication, Table S1). Further database analysis identified seven more partial sequences (six stick insects, one locust) with significant homology to H_V_1 channels, however not or only partially covering the signature motif RxWRxxR (Table S1B).

Within the 18 full‐length clones, sequence identity between species from different polyneopteran orders were 42%–55% within the core region of the channels (S1–S4, 63%–75% homology). Within a polyneopteran order, sequence identity was > 80% (Table S2). All sequences have the typical four‐transmembrane structure comparable to other known H_V_1 channels: short loops between the transmembrane region (8–16 amino acids) and rather short C‐ and N‐terminal domains (~ 50 amino acids). Sequence length varied between 211 and 273 amino acids in total.

**Fig. 1:**
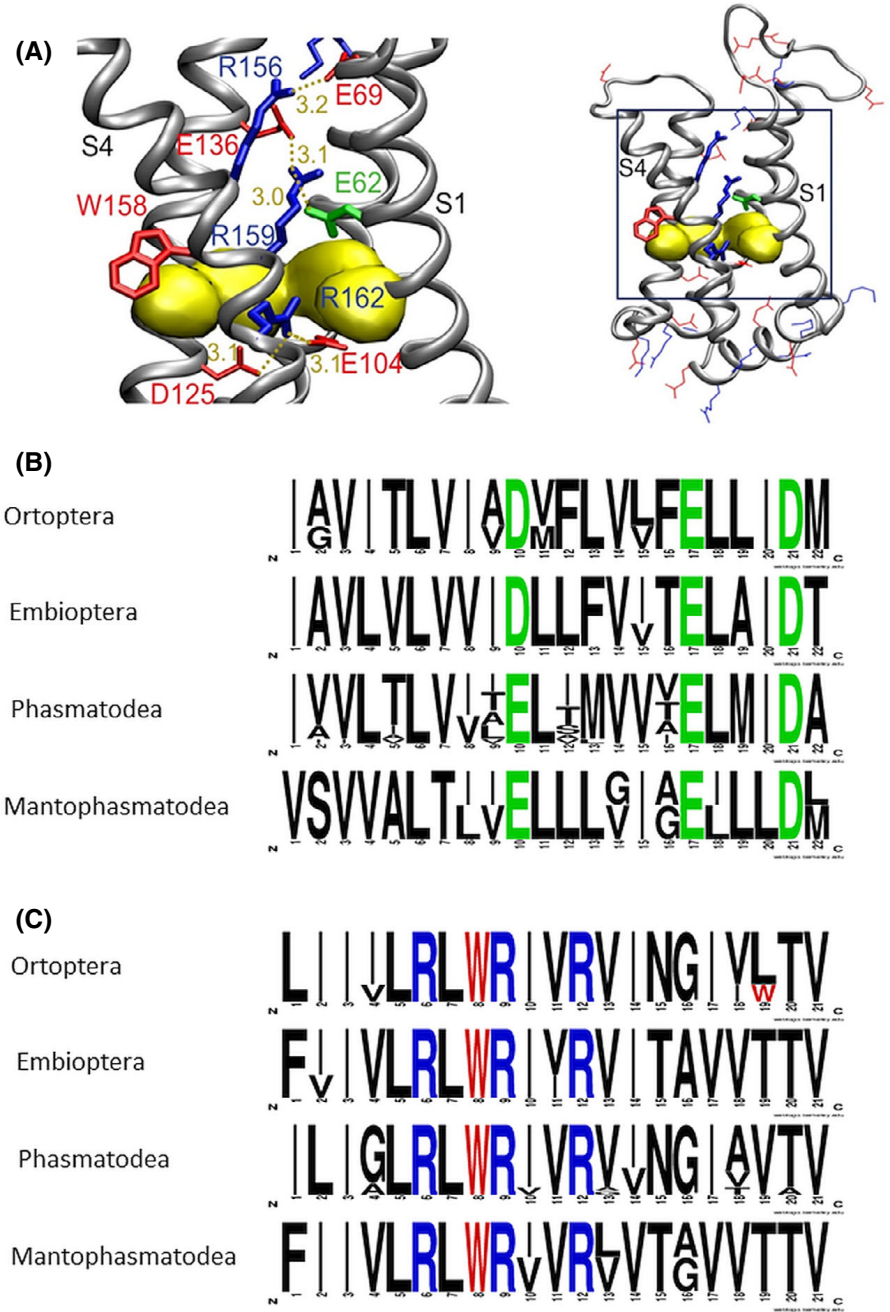
Structure and homology of polyneopteran H_V_1 channels. (A) Structural model of the transmembrane domain (right) and interactions found in the pore (left) in an activated (open) state of *Extatosoma tiaratum*, EtH_V_1. The three voltage‐sensor arginines in S4 are shown in blue, the glutamate SF (in S1) in green, and the conserved tryptophan in red sticks. Other arginine and lysine residues are depicted in blue, aspartate and glutamate residues in red lines. A hydrophobic gasket, depicted as yellow surface, is formed by the side chains of V59, F101, V128 and I129 that separates the inner and outer aqueous vestibules. The network of stabilizing interactions shown as gold dots (distances in Å) is similar to that in human H_V_1 (hH_V_1). R1, here R156, interacts with a glutamate residue, E69 (E119 in hH_V_1), R2 (R159) with the SF E62 (D112 in hH_V_1) and E136 (D185 in hH_V_1), and R3 with E104 (E153 in hH_V_1) and D125 (D174 in hH_V_1). (B and C) Sequence logo representation of H_V_1 S1‐ (B) and S4‐domains (C) showing the amino acid frequencies at respective positions of four different orders of polyneopteran insects. The first green residues shown (position 10 in B) belong to the SF; the voltage‐sensor arginine residues are highlighted in blue and the conserved S4 tryptophan residue in red. Number of sequences used for analysis in S1, S4; respectively: Orthoptera (*n* = 3, 4), Embioptera (*n* = 3, 3), Phasmatodea (*n* = 15, 18) and Mantophasmatodea (*n* = 2, 2).

A striking sequence variation is found within the SF in the S1 segment. In most known H_V_1 channels, a negatively charged aspartate residue (D112 in human H_V_1) is important for proton selectivity. A similar aspartate is found in all orthopteran, mantodean and embiopteran sequences. In phasmatodean and mantophasmatodean sequences however, this aspartate is replaced by an also negatively charged glutamate residue (Figs 1 and S2). A similar exchange (D to E) within the S1 selectivity filter has been artificially generated by different mutagenesis projects [11, 17, 20, 21, 32], resulting in more negative activation, speeding up of activation kinetics but maintaining proton selectivity. Here, for the first time, we show that a glutamate residue occurs naturally at the SF position.

### Do any hemipteran or holometabolan insects harbour a H_V_1 channel homolog?

Despite being overrepresented in protein and nucleotide databases, analysis of all TSA and genomic databases of hemipteran and holometabolan insects revealed only seven TSA sequences encoding the S4 signature motif. The genomic sequence data (mainly from Diptera) showed absolutely no evidence of the presence of an H_V_1 homolog in Hemiptera and Holometabola. A closer look at the respective TSA sequences showed that none of them has high homology to known hexapodan H_V_1 homologs, but show strong homology to fungal H_V_1 sequences (in six cases) and to Chelicerata (one case). Therefore, all identified putative H_V_1 channels within these insect orders are likely due to parasitic contamination of the animal sample investigated (Table S1C). Indeed, especially fungal contaminations are easily uncovered by sequence analysis of H_V_1 homologs, as the third arginine of the RxWRxxR motif is usually mutated to a lysine residue in fungus. We conclude that there is no evidence for the presence of H_V_1 channel homologs in Hemiptera or Holometabola.

### Structure of the *Extatosoma tiaratum* H_V_1 channel (EtH_V_1)

For further characterization, we selected the H_V_1 channel of the stick insect *Extatosoma tiaratum* (EtH_V_1, GenBank Acc. No. GAWG01024136). EtH_V_1 is 236 amino acids (aa) in length, possesses the usual four transmembrane regions and 52 aa N‐terminal and 65 aa C‐terminal intracellular domains. This sequence harbours the phasmatodean‐specific glutamate (E62) as SF in S1 and a typical S4 voltage sensor. Within the core segment, S1–S4 EtH_V_1 is 33% identical and 63% homologous to human H_V_1. Fig. 1A depicts a homology model of EtH_V_1 in open‐state displaying the relative position of relevant amino acids. Both, distances and stabilizing interactions between charged amino acids are in agreement with the human H_V_1. Fig. 1B,C presents the alignment of the S1 (B) and S4 segments (C) of different polyneopteran H_V_1, showing high preservation of the RxWRxxR voltage‐sensor motif and the Asp to Glu natural variation in the SF. A full‐sequence alignment of EtH_V_1 with sequences from other polyneopteran, basal hexapodes and human H_V_1 is shown in Fig. S2.

### Tissue expression of EtH_V_1

**Fig. 2:**
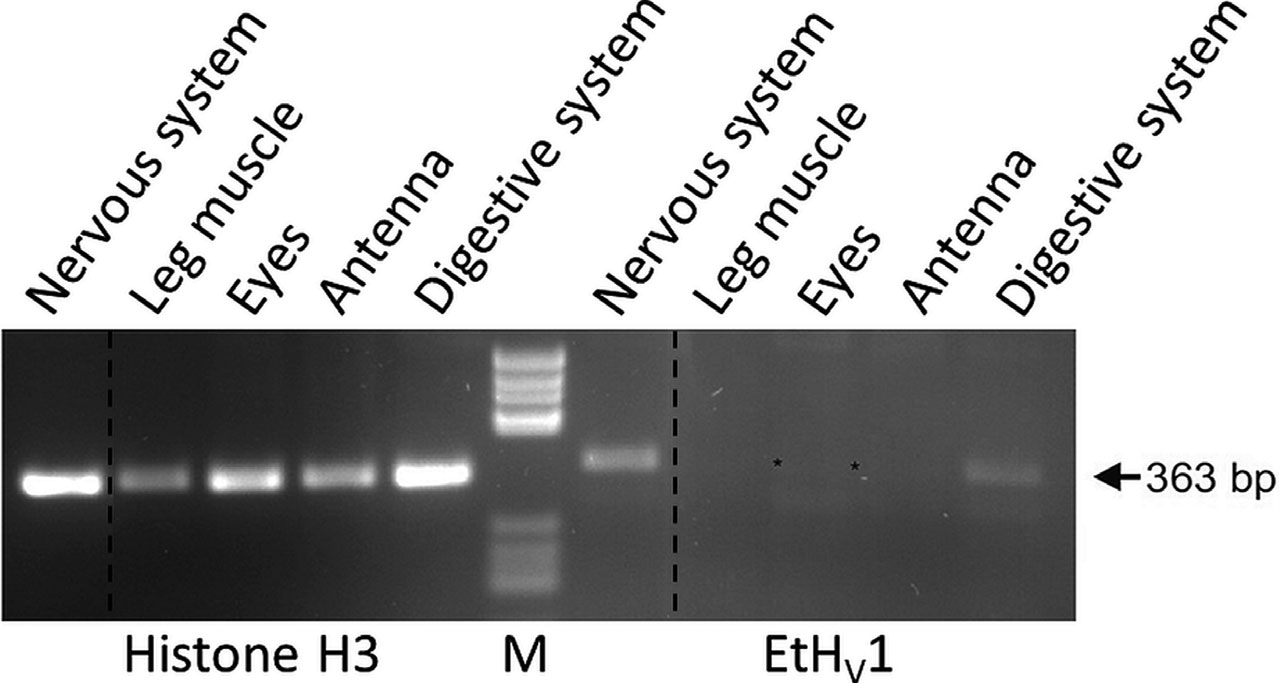
Expression of EtH_V_1 in different *Extatosoma tissues*. Electrophoretical analysis of RT‐PCR samples of the expression of EtH_V_1 and as a control of histone H3 on a 2% agarose single‐gel is shown. EtH_V_1 PCR product is indicated by an arrow; very weak bands are additionally marked by asterisks. Dashed lines indicate the positions where single lanes were deleted due the exclusion of a single sample from this study on demand of the journal reviewers. Marker: pBR322/*Hae*III.

An RT‐PCR analysis of five different tissues isolated from two animals showed strongest expression in the nervous system as a conglomerate of all *Extatosoma* ganglia. Moderate expression was found in the digestive system and weak expression was detected in eyes, whereas no clear expression could be detected in muscle and antenna. In Fig. 2, an agarose gel of the RT‐PCR is shown; the 363 bp EtH_V_1‐PCR product is indicated. As a positive control, *Extatosoma* histone H3 expression was detected in all five tissue samples. PCR products from ganglia and digestive system were verified by DNA sequencing.

### Electrophysiological characterization of EtH_V_1

*Extatosoma* EtH_V_1 was expressed as a GFP fusion protein in tSA cells and was distinguishably localized in the cell membrane when detected under fluorescence. Transfected cells had a capacitance of 9.62 ± 2.03 pF (mean ± SD, *n* = 9 cells) and presented a mean conductance density of 1.07 ± 0.44 nS·pF^−1^ (mean ± SD, *n* = 9 cells), demonstrating reliable expression levels.

Typical proton selective currents were detected during patch‐clamp experiments. Consistent with reports of other species [1, 2, 3, 4, 11, 33, 34], robust H^+^ currents presented threshold potential, *V*_thres_, and time‐dependent behaviour in the order of seconds. The time course of currents has sigmoidal shape which has previously been attributed to the dimeric nature of H_V_1 [22, 35]. After a short delay, currents rise exponentially during membrane depolarization and large tail currents appear at repolarization steps.

**Fig. 3:**
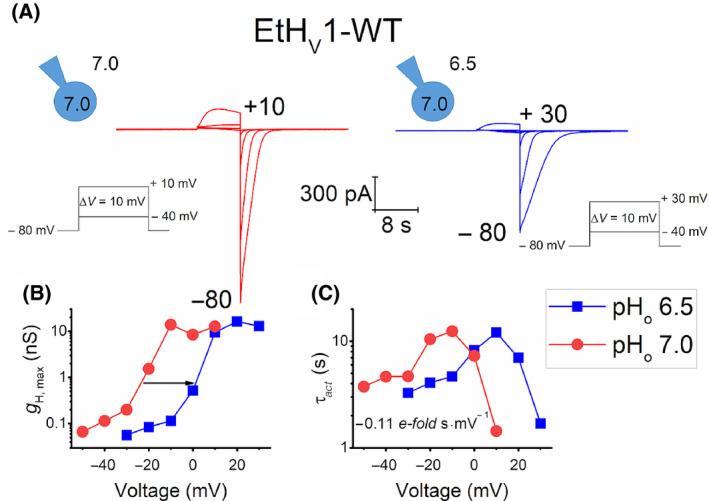
EtH_V_1 presents voltage‐ and pH‐dependent gating. (A) Whole‐cell patch‐clamp measurement of a cell expressing EtH_V_1 at pH_o_ 7.0 (left, red traces) and 6.5 (right, blue traces). Families of pulses were obtained by depolarization of the cell membrane in 10 mV increments from −40 mV to the shown voltage. The holding potential for both pH conditions was −80 mV and the pH_i_ was 6.5. Scale bars apply for both families of pulses. (B) Maximal conductance–voltage plot of the families of pulses shown in (A). EtH_V_1 adjusts its *g*_H_ according to a negative pH gradient, ∆ pH = pH_o_ − pH_i_, shifting to more positive voltages (black arrow). (C) Activation kinetics plot showing a voltage dependence of τ*_act_* of – 0.11 *e‐fold* s·mV^−1^.

Figure 3A depicts an example of a whole‐cell patch‐clamp measurement of EtH_V_1 in two different pH conditions. The amplitude of currents is time‐dependent and increases with every depolarizing step, clearly indicating voltage‐dependent activation. Large and relatively slow tail currents are also seen once the channel deactivates as consequence of repolarization of the cell membrane (e.g. *V*_hold_ = −80 mV).

In common with other H_V_1 channels, *g*_H_ of EtH_V_1 is also regulated by the pH gradient across the membrane, ∆pH (pH_o_ − pH_i_). When ∆pH increases (∆pH > 0) or decreases (∆pH < 0), EtH_V_1 adjusts its *g*_H_ to more positive or to more negative potentials accordingly. Figure 3B shows a clear rightward shift of the conductance – voltage relationship, *g*_H_‐*V*, of ~ 25 mV once external pH (pH_o_) was diminished from 7.0 to 6.5 (black arrow). The same behaviour was detected in inside‐out patches where pH_i_ was exchanged to generate the same ∆pH = −0.5 (Fig. S3).

The effect of *g*_H_‐*V* change can also be seen on EtH_V_1 activation kinetics, τ*_act_* (Fig. 3C). The voltage dependence of τ*_act_* of EtH_V_1 is represented by a slope of −0.10 ± 0.02 *e‐fold* s·mV^−1^ (mean ± SD, *n* = 6) which renders into 10 mV/*e‐fold* change.

**Fig. 4:**
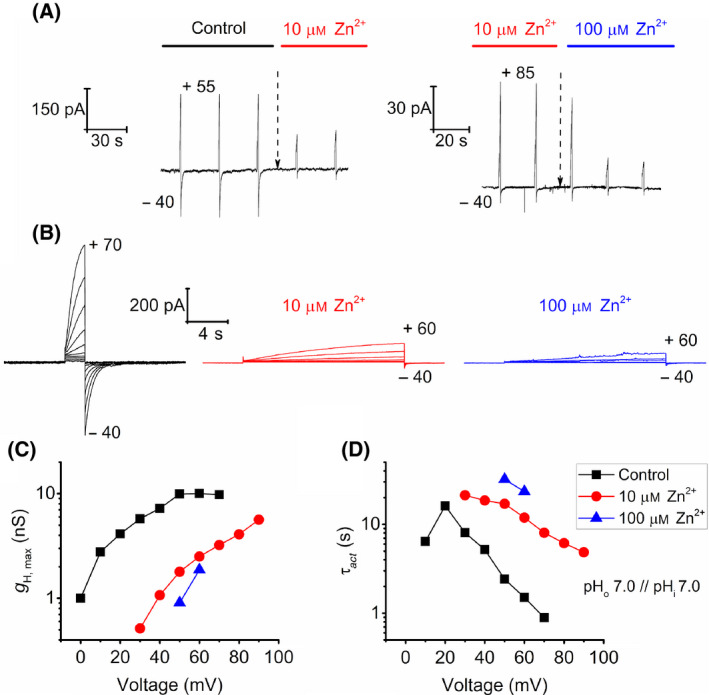
EtH_V_1 H^+^ currents are clearly inhibited by external addition of Zn^2+^. (A) Inhibition of proton currents by Zn^2+^ addition during depolarizing pulses at pH_i_ = 7.0 and pH_o_ = 7.0. Time points where initiation of bath exchange took place are indicated by dashed arrows. *Left panel:* Bath exchange of a free Zn^2+^ solution (Control) to a solution containing 10 μm Zn^2+^. The test pulse (*V*_test_) and the holding potential (*V*_holding_) were +55 mV and −40 mV, respectively. *Right panel:* solution exchange from 10 μm Zn^2+^ to 100 μm Zn^2+^ conditions. *V*_test_ = +85 mV and *V*_holding_ = −40 mV. (B) Whole‐cell recordings of the same cell at 0 (black traces), 10 (red traces) and 100 µm Zn^2+^ (blue traces) at pH_i_ = 7.0 // pH_o_ = 7.0. Depolarizing pulses were applied in 10 mV steps from the holding potential, −40 mV, to the potential showed in each family. (C) *g*_H_ as function of voltage in 0 μm (black squares), 10 μm (red dots) and 100 μm Zn^2+^ (blue triangles) conditions. *g*_H_‐*V* relationships shift rightward with the increase of [Zn^2+^]. (D) Slowing of the activation kinetics due to rise of external [Zn^2+^]. Legend and pH conditions depicted apply also for C.

We further tested the classical proton channel inhibitor, zinc, on EtH_V_1. Figure 4 shows how Zn^2+^ affects EtH_V_1 H^+^ currents in the same cell. The divalent cation drastically reduces the amplitude and kinetics of proton currents activation. In our experiments, we increased [Zn^2+^] from 0 (Control) to 10 and 100 μm. During test pulse protocols, H^+^ activation and tail currents reduce their amplitude once zinc is added to the bath solution (Fig. 4A). Figure 4B depicts families of pulses under the three different zinc conditions in a whole‐cell configuration. Two main effects are shown by the families of pulses: a reduction of activation currents at the same depolarization and a slowing of activation kinetics. In agreement with other H_V_1 studies [5, 25, 34, 35, 36], *g*_H_‐*V* curves shift rightwards along the voltage axe (Fig. 4C), and τ*_act_* becomes slower once [Zn^2+^] increases (Fig. 4D). Both are the two main effects of zinc inhibition on proton channels. The data demonstrate that EtH_V_1 is sensitive to the classical proton channel inhibitor, zinc, in a micromolar range.

### Proton selectivity and pH‐dependent gating of EtH_V_1

The reversal potential of EtH_V_1 was analysed in a pH_o_ range between 5.5 and 7.0, and pH_i_ 6.5–7.0. Because activation of EtH_V_1 was negative to *V*_rev_ for most of the cases, *V*_rev_ was determined directly as the zero current in a family of depolarizing pulses.

**Fig. 5:**
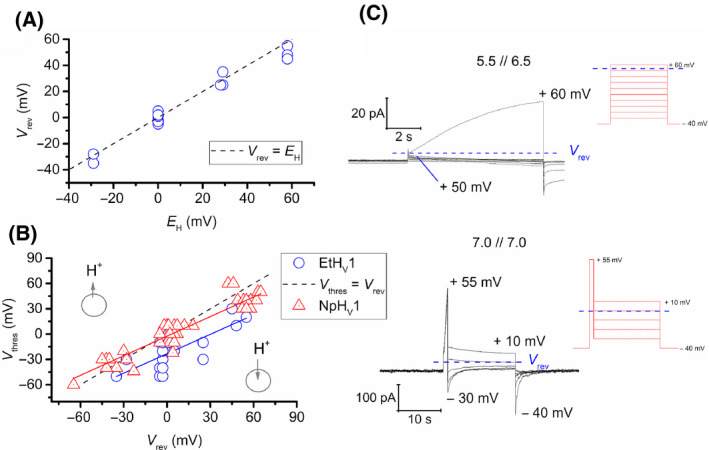
EtH_V_1 is a proton channel that activates more negative than NpH_V_1. (A) Measured reversal potentials, *V*_rev,_ are plotted against the *E*_H_. The dotted line represents equality between *V*_rev_ and *E*_H_, indicating perfect proton selectivity. (B) Comparison of the pH dependence of gating between EtH_V_1 and NpH_V_1. Threshold potential, *V*_thres_, are plotted versus the reversal potential for both, EtH_V_1 (blue circles) and NpH_V_1 (red triangles), in a voltage range from −70 mV to + 70 mV. Dotted line represents equality between *V*_rev_ and *V*_thres_. Blue and red solid lines show the linear regression of data from EtH_V_1 and NpH_V_1, respectively. For the same voltage range, NpH_V_1 presents a pH‐dependent gating equal to *V*_thres_ = 0.77 *V*_rev_ – 2.4 mV (*n* = 41); meanwhile, EtH_V_1 shows a more negative activation defined by *V*_thres_ = 0.77 *V*_rev_ – 23 mV. *n* = 16 (9 cells), pH_i_ was 6.5 or 7.0, and measurements made in a pH_o_ range from 5.5 to 7.0. (C) *Upper recording*: activation of proton currents (the conductance activated negative to *V*_rev_) in a whole‐cell patch‐clamp configuration show a *V*_rev_ between +50 and +60 mV when pH_i_ = 6.5 and pH_o_ 5.5, accordingly with a predicted *E*_H_ of +58 mV in the same pH conditions. Pulses were applied in 10 mV increments from the holding potential (−40 mV) to +60 mV. *Lower recording*: Tail current records of a patch at symmetrical pH_i_ // pH_o_ 7.0 indicating a *V*_rev_ close to 0 mV. Test pulses were applied in 10 mV increments after a depolarizing pulse (+55 mV) from the holding potential (−40 mV) to +10 mV.

The recorded values follow accurately the predicted Nernst potential for proton conduction, *E*_H_, indicating that EtH_V_1 is highly proton selective (Fig. 5A). Deviations of *V*_rev_ values from *E*_H_ are a consequence of incomplete pH_i_ control even though high pH buffer concentrations were used, for example strong depolarization causes H^+^ depletion that increases pH_i_. Increase in internal proton concentration, [H^+^]_i_, and the consequent drop of internal pH (pH_i_) causes divergences between measured *V*_rev_ and calculated *E*_H_. A rise in [H^+^]_i_ is provoked by consistent inward H^+^ currents during channel’s activation, when *V*_thres_ < *V*_rev_, or by large tail currents observed during membrane repolarization.

In H_V_1, voltage and pH modulate the channel’s gating. To evaluate the pH dependence of gating of EtH_V_1, we applied the ‘threshold versus reversal’ strategy previously used in other studies [2, 11, 37]. The approach consists of determining the reversal and threshold potential at a wide range of pH to obtain an equation of the form *V*_thres_ = *slope*·*V*_rev_ + *offset*. We measured *V*_rev_ and *V*_thres_ at pH ranges from 5.5 to 7.0 applying different ∆pH (Fig. 4B). In a total of 16 determinations, data permit to define the voltage dependence of EtH_V_1 as:

(1) $$V_{\text{thres}}=0.77\;V_{\text{rev}}‐23\;\text{mV}$$

Interestingly, EtH_V_1 and NpH_V_1 present the same voltage dependence of gating translated to a slope of 0.77 *V*_thres_/*V*_rev_. Nevertheless, a major difference in the offsets of both channels can be seen. *Extatosoma* is more negatively activated (−23 mV) than *Nicoletia* (−2.4 mV). The dotted line in Fig. 5B represents equality between *V*_thres_ and *V*_rev_. Data located under the dotted line stand for inward H^+^ conduction, while data points above the dotted line represent outward H^+^ currents. By definition, if *V*_thres_ is positive to *V*_rev_, H_V_1 conducts protons outwards, alkalinizing the cytosol. In opposition to this, threshold values negative to *V*_rev_ show inwardly directed proton currents. Thus, Fig. 5B enables the fast determination of the proton currents direction. In the whole investigated pH range, EtH_V_1 activation is negative to *V*_rev_. EtH_V_1 permits proton influx. In contrast, NpH_V_1 activation is ~ 20 mV more positive and permits proton extrusion, while mostly preventing inward H^+^ flux.

## Discussion

### Distribution among hexapodans

**Fig. 6:**
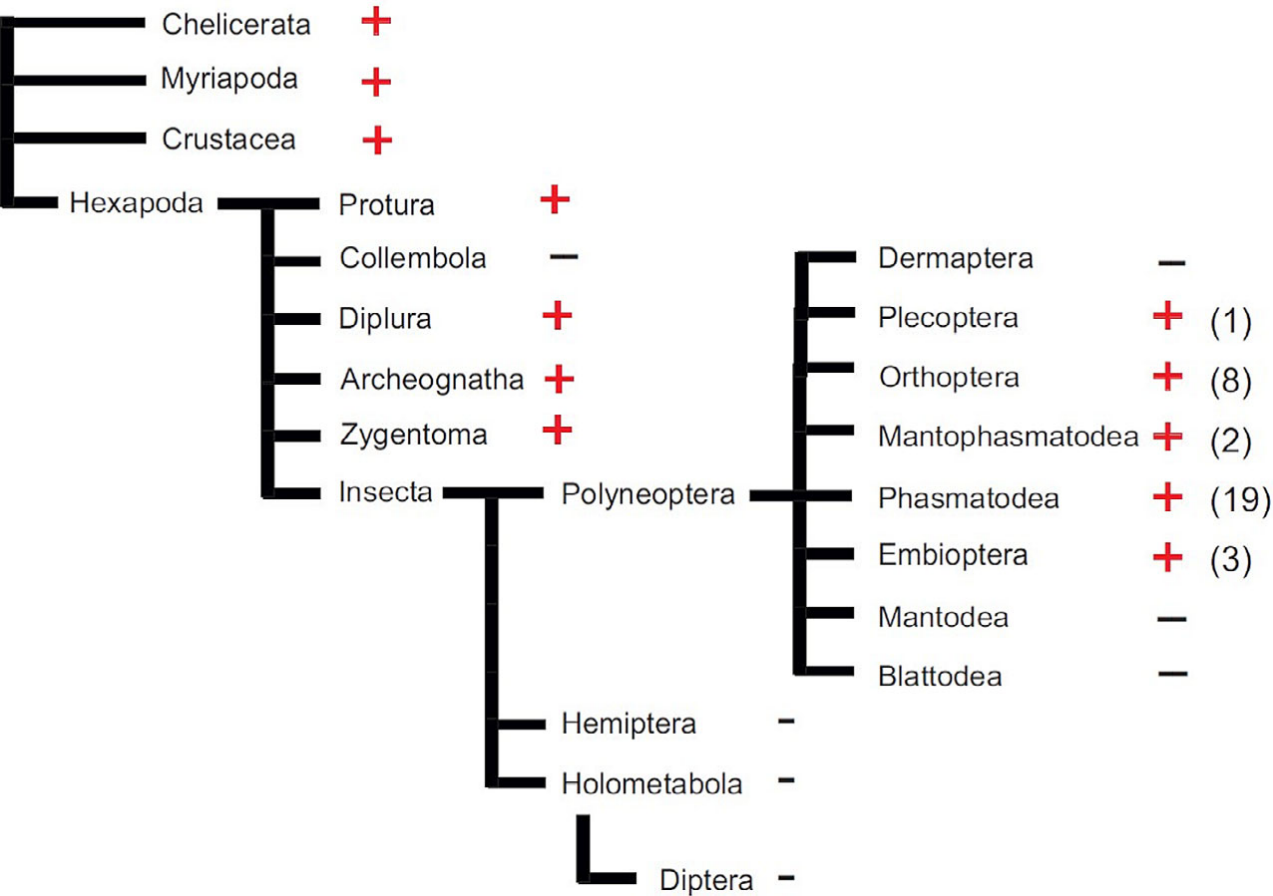
Schematic representation of the phylogenetic relationships among major hexapodan lineages. The presence or absence of a typical H_V_1 homolog within TSA and genomic databases is indicated. For polyneopteran species, the number of identified putative H_V_1 channels is shown. The cladogram is based on the most recent insect phylogeny [31].

Proton channels are unique members of the voltage‐gated ion channel superfamily, as they are represented in most species by a single gene or by no gene at all. This indicates that H_V_1 offers an evolutionary advantage over some species, whereas other species may dispense an H_V_1 homolog. The common ancestor of Hexapoda, Crustacea, Myriapoda and Chelicerata clearly possesses a single H_V_1 gene. Figure 6 shows a schematic representation of the phylogenetic relationships among major hexapodan lineages. A correlation with the presence or absence of a typical H_V_1 homolog suggested, that the H_V_1 gene was lost several times within the hexapodes: (a) within Colembola, (b) within the common ancestor of the phylogenetically more derived insects (Hemiptera and Holometabola), and (c) once or twice within different polyneopteran orders. Considering Polyneoptera as monophyletic group, it is obvious that the common ancestor of the sister groups Mantodea and Blattodea lost an H_V_1 homolog. The absence of H_V_1 within these orders is very likely since sequence coverage of these orders is high. Furthermore, it is extremely unlikely that in all species analysed the putative H_V_1 homolog has simply been missed by sequencing, instead of been lost during evolution.

During this study, nine TSA sequences were identified with significant homology to known fungal sequences, with up to 99% identity. These clones undoubtedly represent sample contaminations. The 1KITE project and other related TSA studies provide us with a huge amount of transcriptomal sequence data [31]. Despite the overall data being of very good quality and sequence coverage also being high (depending somewhat on the species analysed), a major drawback is sample contaminations from insect parasites, mainly fungus. As whole insects were analysed by TSA studies, such contaminations cannot be excluded within the first sequence drafts. Indeed, algorithms were used to eliminate such noninsect sequences from the dataset; however, some contaminations are still found. Actually, from ten insect TSA database entries representing clear contaminations, only three were subsequently removed by the 1KITE staff (~ 30% of all contaminated TSA entries, ~ 50% of contaminated 1KITE TSA data).

### Glutamate as SF of EtH_V_1

The unusual glutamate residue in the S1 selectivity filter (E62 in *Extatosoma*) is found only in the two closely related polyneopteran orders of Phasmatodea and Mantophasmatodea. Indeed, homologous positions at the SF have been characterized in detail by site‐directed mutagenesis in human [17], dinoflagellate [2] and also in the Zygentoma *Nicoletia phytophila* H_V_1 [11]. Asp to Glu substitutions at the SF were done considering that both residues are negatively charged at physiological conditions. The investigations proved that H_V_1 is still proton selective once a glutamate is present at the SF position in S1 [2, 11, 17]. On the other hand, the extreme proton selectivity is lost once Asp is mutated to a neutral amino acid, for example alanine (Ala), making the channel also permeable to anions [2, 11, 17]. A potential mechanism explaining the necessity of a negatively charged amino acid at this position has been reported by Dudev et al. [18]. They analysed the selectivity mechanism of H_V_1 applying a quantum‐based model. In the open state of the channel, the SF is composed of a salt bridge interaction between the Asp and the second or third Arg of the voltage‐sensor motif RxWRxxR, in a constricted part of the channel. When placed in‐between the Asp‐Arg SF, H_3_O^+^ enables protonation of the Asp, breaking the electrostatic Asp‐Arg interaction in an energetically favourable process. Later, the gained H^+^ is transferred from the protonated aspartate (AspH) to a neighbouring nucleophile and the Asp‐Arg interaction restored, allowing other protons to initiate the process again. In this way, protons can travel through the SF of a H_V_1. Other competing ions as Cl^−^ and Na^+^ are repelled by the residue bearing the same charge (e.g. Asp for Cl^‐^ and Arg for Na^+^) or trapped by the residue of opposite charge and cannot cross though the Asp‐Arg SF [18].

In voltage‐gated proton channels (H_V_1), the existence of a negatively charged residue at the SF is mandatory for proton selectivity and E62 in *Extatosoma* meets this requirement. The pk_a_ of the glutamate side chain is ~ 4.25, which enables the residue to be deprotonated at pH > 5.0 and to remain negatively charged as consequence. This would permit EtH_V_1 to have a SF composed of a Glu‐Arg interaction working in a similar manner as the Asp‐Arg selectivity mechanism.

The selectivity of an ion channel can be represented by the Goldman–Hodgkin–Katz, GHK equation (Eqn 2):

(2) $$V_{\mathrm{rev}}=\frac{\mathrm{RT}}{F}\;\ln\left(\frac{\sum_{i}^{n}P_{M_{i}^{+}}{\left[M_{i}^{+}\right]}_{out}+\sum_{j}^{m}P_{A_{j}^{‐}}{\left[A_{j}^{‐}\right]}_{in}}{\sum_{i}^{n}P_{M_{i}^{+}}{\left[M_{i}^{+}\right]}_{in}+\sum_{j}^{m}P_{A_{j}^{‐}}{\left[A_{j}^{‐}\right]}_{out}}\right)$$

(3) $$E_{H}=\frac{\mathrm{RT}}{\mathrm{zF}}\;\ln\frac{{\left[H^{+}\right]}_{\mathrm{out}}}{{\left[H^{+}\right]}_{\mathrm{in}}}$$

where *V*_rev_ = reversal potential (V or J·C^‐1^); *R* = ideal gas constant (J·mol^−1^·K^−1^); *T* = temperature (K); *F* = Faraday constant (C·mol^−1^); *P_ion_* = permeability for that ion (m·s^−1^); *z* = ion charge; *E*_H_ = Nernst potential for protons; *[ion]_out_* = extracellular concentration of that ion (M); *[ion]_in_* = intracellular concentration of that ion (M).

In our experiments, protons were in a concentration from 0.1 μm (pH 7.0) to 3.12 μm (pH 5.5), between four to five orders of magnitude lower than the concentrations of the main ions TMA^+^ and CH_3_SO_3_^−^ (90–125 mm). Despite this great disproportion for protons, all measured *V*_rev_ follow nernstian behaviour of protons (Eqn 3). Small variations are mainly consequence of an imperfect control of the pH_i_. Depletion and accumulation of H^+^ due to high depolarization and robust inward H^+^ currents, respectively, are a common source of error while measuring H_V_1 in the whole‐cell patch‐clamp configuration. In this configuration, the accuracy of the control of cytosolic pH is limited by the diffusion rate of the buffer between the pipette and the cell. This diffusion exchange lasts from seconds to even minutes [38]. In our experiments, we try to circumvent this problem by shortening pulses at very positive voltages and/or increasing resting times between pulses. Our data specify EtH_V_1 as a proton selective channel.

### EtH_V_1 is inhibited by Zn^2+^

Inhibition of proton currents by external addition of zinc is considered one of the main characteristics of H_V_1. We tested the response of EtH_V_1 to external Zn^2+^ in micromolar range concentrations. Our experiments recorded inhibition of H^+^ currents at 10 μm Zn^2+^ which was augmented once [Zn^2+^] further increased (Fig. 4A,B). The inhibitory effect is better seen as a shift of the *g*_H_‐*V* relationship and as slowing of the kinetics of activation (Fig. 4C,D). The tendency is similar to other tested H_V_1 [5, 25, 34, 35, 36]. The mechanism of inhibition of proton channels by zinc is still an ongoing discussion; nevertheless, several studies have identified some of the amino acids involved.

Mammalian proton channels possess two identified Zn^2+^ binding sites composed exclusively of external His residues [3, 36]. The first one is located at the top of S2 alpha helix and the second is placed in the S3‐S4 loop (H140 and H193 in the human H_V_1). Substitution of these two His residues to Ala renders the channel zinc insensitive [3, 35]. In contrast, proton channels of further species show more diversity. For example, the other characterized insect proton channel, NpH_V_1, conserves a His residue (H92) at the same relative position of H140 of the human channel but presents a variation to Asp (D145) in the second biding site, H193. A detailed zinc inhibition analysis demonstrated that in the case of NpH_V_1, the main inhibitory effect is caused by zinc binding to the first position, H92, with minimal participation of the second binding site, D145. The inhibition of NpH_V_1 by Zn^2+^ is smaller than in human and rat channels [25]. Nevertheless, zinc sensitivity of *Nicoletia* is greatly increased by mutation of D145 to His and completely abolished once both amino acids are mutated to Ala, similar to mammalian H_V_1 channels [25]. It seems that histidine residues at these two precise locations of H_V_1 are important for high zinc sensitivity.

Interestingly, the putative Zn^2+^ binding sites of EtH_V_1 consist of a lysine residue (Lys91) and of an aspartate residue (Asp144) at the first and second positions, respectively (see alignment of Fig. S2). A Lys residue at the first zinc binding site is a peculiarity of EtH_V_1 but remarkably common to all identified polyneopteran H_V_1. However, this noteworthy difference is lost in other orders as Zygentoma, Diplura, Protura and Archeognatha, whose H_V_1 have the regular His residue also present in mammal channels. On the other hand, the second putative binding position (Asp144) on the S3–S4 linker of *Extatosoma* is preceded by two consecutive histidine residues, His142 and His143, which are potentially coordinating Zn^2+^. The same –His‐His‐Asp‐ pattern is shared with other phasmatodean H_V_1 homologs: SsH_V_1, RaH_V_1 and MeH_V_1 (Fig. S2). Yet, other phasmatodean species (*Aretaon asperrimus* and *Peruphasma schultei)*, the Embioptera *Aposthonia japonica* and the Archeognatha *Pedetontus okajimae*, present in contrast only one His residue next to the Asp of the S3–S4 linker.

Investigations in *Nicoletia* confirmed the dimeric nature of an insect proton channel and the possibility of zinc binding at the interface of both monomers [25]. Coordination of zinc in‐between H_V_1 subunits has also been suggested in inhibition studies of the human channel [35, 39]; hence, EtH_V_1 stoichiometry might be a factor to be considered.

Structural differences between Zn^2+^ binding sites among species also generate different zinc sensitivities. Therefore, potency of Zn^2+^ on H_V_1 can be related to the surrounding [Zn^2+^] and the function of proton channels in the organisms. Thus, low zinc concentration in human and mouse serum ranges from 13 to 20 μm [40] and the respective H_V_1 shows consequently higher zinc sensitivity than in other species, for example *Nicoletia phytophila* (insect), *Ciona intestinalis* (sea squirt) and *Helisoma trivolvis* (snail) [34]. The sensitivity to Zn^2+^ revealed by the animal model *Danio rerio* (zebrafish) is even lower, which associates with the considerably higher zinc concentrations in the serum of the animal (~ 150 μm) [40]. Unfortunately, there are no data available determining the concentration of zinc in the haemolymph of *Extatosoma*.

Further studies, including the pH dependence of Zn^2+^ inhibition, site‐directed mutagenesis of putative binding sites and the analysis of the channel oligomerization are still necessary to address the nature of zinc inhibition of polyneopteran H_V_1 channels.

### EtH_V_1 has conventional pH dependence of gating with strong voltage‐dependent kinetics of activation

The pH dependence of gating in EtH_V_1 is described by a slope of 0.77 *V*_thres_/*V*_rev_ which translates into a shift of the conductance–voltage relationship of ~ 45 mV per unit of ∆ pH. The value is similar to other proton channels (Table 1) with exception of *Helisoma trivolvis* which reports an anomalous pH dependence of gating [34]. Moreover, the two insects *Extatosoma* and *Nicoletia* have identical pH dependence of gating for the same voltage range (Fig. 5B). The conformity of the pH‐dependent gating of H_V_1 of different species indicates a common pH sensing mechanism which to date is still unknown. However, differences in the offsets among different species are evident. Our analysis shows an offset of −23 mV for EtH_V_1. A negative offset of the *V*_thres_‐*V*_rev_ relationship reflects an early activation which permit protons to flow from the external solution into the cell. Along the whole pH range tested, EtH_V_1 conducts H^+^ inwards consistently. The results contradict the more positive activation of NpH_V_1 and mammalian channels, whose physiological roles relate to elimination of excessive cytosolic acidification [37] and compensation of electrical charges during the respiratory burst of phagocytes [41]. The negative activation of EtH_V_1 is in contrast more similar to kH_V_1 from the dinoflagellate *Karlodinium veneficum* [2]. In dinoflagellates, inward H^+^ currents acidify the interior of membrane specialized compartments (scintillons) which triggers bioluminescence [5, 34]. Hypothetically, EtH_V_1 in *Extatosoma* plays a role in an acidification process or in the generation of action potentials. To analyse the activation of EtH_V_1 in more detail, we measured the voltage dependence of EtH_V_1 kinetics. We applied linear regressions to τ*_act_* – *voltage* plots at different pH. Results show that EtH_V_1 has a stark steepness of the τ*_act_*–*V* relationship of 10.0 mV/*e*‐fold change, similar to the snail channel HtH_V_1, and much stronger than mammalian channels which values vary between 40 and 72 mV/e‐fold change [37] (Table 1). In an attempt to evaluate if glutamate as SF changes the free energy to open the channel, we decided to analyse our previous data from Nicoletia. NpH_V_1‐D66E mutant increases the voltage dependence of τ*_act_* from 29.4 to 21.3 mV/*e*‐fold change (pH_i_ = 5.5, pH_o_ = 5.5; *n* = 3).

**Table 1:** Comparison of the pH and voltage dependence of gating between different H_V_1.

H_V_1	Species	Slope (*V*_thres_/*V*_rev_)	Offset (mV)	τ*_act_* voltage‐dependence (mV/e‐fold change)	Reference
hH_V_1‐GFP	*H. sapiens*	0.82	+13.8	54–58.7^a^	[3, 37]
hH_V_1‐D112E‐GFP	*H. sapiens*	0.59	n.d.	n.d.	[32]
RnH_V_1 (native)	*R. norvegicus*	0.76	+18	46‐64	[36, 52]
Endogenous H_V_1	Various (15 cells)	0.79	+23	n.d.	[37]
kH_V_1‐GFP	*K. veneficum*	0.79	−37	n.d.	[2]
HtH_V_1‐GFP	*H. trivolvis* ^b^	1.03^c^	n.d.	13.0	[34]
NpH_V_1‐GFP	*N. phytophila*	0.81	−3.41	29.4	[11, 25]
NpH_V_1‐D66E‐GFP	*N. phytophila*	‐	n.d.	21.3	This work
EtH_V_1‐GFP	*E. tiaratum*	0.77	−23	10.0	This work

^a^ Reported values for native H_V_1 in human neutrophils and eosinophils [37].

^b^ H. trivolvis reported an anomalous voltage‐dependent gating in comparison to other H_V_1, for changes in pH_i_ (15.3 mV·pH^−1^) and in pH_o_ (60 mV·pH^−1^) [34].

^c^ Calculated from the reported value of 60 mV·pH^−1^ (pH_o_) and *E*_H_ = 58 mV·pH^−1^.

n.d., no data available.

Despite EtH_V_1 and HtH_V_1 sharing steeper voltage dependence of activation kinetics, in terms of absolute kinetics at symmetrical pH_o_ // pH_i_, activation of H^+^ currents in EtH_V_1 are more similar to mammalian H_V_1. EtH_V_1 activates in the range of seconds just like H_V_1 of mammals, in contrast to HtH_V_1 which presents τ*_act_* values of few milliseconds [34]. The other hexapod proton channel, NpH_V_1, activates also in the range of seconds [11]. Interestingly, in the human channel, data suggest that a glutamate at the position of the SF speeds up the channel activation kinetics. The hH_V_1‐D112E mutant is ~ 5 times faster than the wild‐type [32] and also shifts *V*_thres_ to more negative potentials [20, 21], indicating a shift of free energy to open the channel.

### Possible physiological role

For a functional analysis of insect proton channels, a detailed cellular expression pattern would be of great importance. So far, only tissue distributions of the H_V_1 expression are available. Compared to the Zygentoma *Nicoletia phytophila*, EtH_V_1 showed a more restricted expression pattern in the different tissues tested. In both, *Nicoletia* and *Extatosoma*, H_V_1 is strongly expressed in the nervous system. Interestingly, no expression in leg muscle was found for *Extatosoma*, despite it being present in leg and body muscle in *Nicoletia*.

Harrison [42] describes several patterns of acid–base regulation in insects. The passive transport of protons through H_V_1 could be related to the pH‐homeostasis maintenance in some of these processes in polyneopteran species.

There are pH differences across the digestive system of some insects. In crickets and grasshoppers (Orthoptera), passive distribution of protons across the midgut epithelium is associated with low pH in the lumen [42]. Coincidently, we found a mild expression of EtH_V_1 in digestive system (Fig. 2).

Discontinuous ventilation of insects generating variations of partial CO_2_ pressure (P_CO2_) is also mentioned. The fluctuations on P_CO2_ during discontinued ventilation change the pH of the haemolymph, for example in grasshoppers, where haemolymph pH correlates with fluctuations of P_CO2_ and the nonbicarbonate buffer values [42]. Nevertheless, these pH variations due to discontinuous ventilation are considered small [42].

Other pH‐homeostasis changes in insects are associated with periods of activity. In general, the increase of activity, for example during flight, is accompanied by the use of anaerobic metabolism that generates acid production. In locust, for example, tracheal and fluid P_CO2_ during flight increases two‐ to threefold in comparison to the resting state [42]. Accumulation of CO_2_ due to insect’s activity translates to a drop of haemolymph pH of ~ 0.2 units for grasshoppers and even to 0.9 for cockroaches (do not express H_V_1) during flight [42]. Despite EtH_V_1 was not found in leg muscle, the haemolymph circulates through the whole body of the animal. Hence, we cannot discard the channel involved in pH regulation of the haemolymph during activity periods.

The pH of the haemolymph of some invertebrates decreases linearly with temperature [43]. Similarly, in orthopteran insects (which do have H_V_1), the haemolymph pH appears to be also dependent on temperature although the temperature–pH relationship loses linearity. Thus, the orthopterans *M. bivittatus* and *S. nitens* are able to keep a constant haemolymph pH at temperatures of 10 °C–25 °C but the value drops with a rate of 0.017 units·°C^−1^ at temperatures higher than 25 °C [42]. Transmembrane acid–base transport controlled by the renal system has been suggested to explain this behaviour [44]. EtH_V_1 could also play an important role in relation to acid regulation.

Another possible physiological role of EtH_V_1 could be related to the sensitivity of chemoreceptors to haemolymph pH. For example, cockroaches (which lack of H_V_1) abdominal pumping rates are regulated by the pH of solutions in contact with the nerve cord [45]. Grasshoppers on the other hand possess H_V_1 and their ventilation rates are unaltered once the haemolymph pH is changed [46].

The EtH_V_1 channel is highly expressed in the nervous system (Fig. 2). Remarkably, H_V_1 was first discovered in snail neurons by Thomas and Meech [47]. Subsequent studies in neurons of other snail species [48, 49] confirmed the existence of H_V_1 presenting τ*_act_* of few milliseconds [50]. The activation of EtH_V_1 is negative to *V*_rev_ in the whole pH range tested. It implies that EtH_V_1 conducts H^+^ inwardly and therefore could depolarize the cell membrane. In the case that H^+^ conductance is dominant in the membrane of neurons under ionic conditions of the animal at certain membrane potentials, small inward currents could effectively depolarize the neuron to action potential threshold. Proton channels of mammals, activating in the order of seconds, restore pH_i_ of small cells after an acid load in the order of tens of seconds because of their surface/volume ratio [50]. However, a role of EtH_V_1 in the generation of action potentials is presumably limited due to its relatively slow activation. In neurons of *Locusta migratoria* (Orthoptera) for example, the times‐to‐peak range from ~ 2 to 10 ms [51]. Our data do not confirm or discard the participation of EtH_V_1 in the generation of action potentials in *Extatosoma*. Further *in vivo* electrophysiological studies in *Extatosoma* neurons are required to evaluate involved conductances and the effects of pH variations on triggering of action potentials.

A striking difference we found between the hexapodans proton channels NpH_V_1 and EtH_V_1 is the more negative opening of the later. Consistently, EtH_V_1 activates approximately 20 mV more negative than NpH_V_1. This means that in comparison with NpH_V_1, activation of EtH_V_1 presents a shift of free energy that favours the close→open transition due to the influence of the membrane potential. EtH_V_1 requires less membrane depolarization to activate. Hypothetically, the natural occurring variation to Glu in the SF of EtH_V_1 might be responsible for it. Site‐directed mutations of Asp112 to Glu in the SF of the human H_V_1 have revealed negative shifts of threshold of activation [20, 21]. Mutations of other amino acids in other parts of the channel also provoke ∆*V*_thres_ to more negative potentials. However, in accordance with a meta‐analysis of mutation studies [32], of all Asp mutants in the SF, only the Asp to Glu mutation shifts *V*_thres_ negatively.

The activation of EtH_V_1 is also negative to *V*_rev_, which translates to an inward H^+^ current that acidifies the cytosol. Teleologically, EtH_V_1 task is related to the acidification of a cell or a cell compartment or maybe alkalinization of the extracellular milieu. Similarly, marine dinoflagellates, whose H_V_1 channels activate also negative to *V*_rev_, use H_V_1 channels to acidify scintillons and trigger bioluminescence [2, 5]. The chemistry of the physiological environment must always be considered. Thus, if [Zn^2+^] is elevated in Extatosoma, then the *V*_thres_ is shifted to positive potentials. Assuming the voltage shift is sufficient to set *V*_thres_ positive to *V*_rev_, in this case, EtH_V_1 functions similar to most known H_V_1 and extrude protons out of the cell.

Embioptera (Asp in SF) and Phasmatodea (Glu in SF) belong to sister branches with a common ancestor [12]. Interestingly, their cousin branch Mantophasmatodea also has a Glu in the SF [12]. Perhaps the answer to the function of Glu as SF and its relationship with the physiology of the insect lies on the physiological differences between those polyneopteran orders.

## Conflict of interest

The authors declare no conflict of interest.

## Author contributions

GC and BM designed and performed patch‐clamp experiments. CD performed GenBank search and RT‐PCR, sampled RNA. CJ generated structural models. AF cloned DNA. GC, CD and BM analysed and interpreted data. GC, CD and BM wrote the manuscript. All authors approved the manuscript.

## Supporting information


**Table S1.** List of all identified H_V_1 homologs compiled from TSA files and correspondent GenBank accession number.
**Table S2.** Sequence identity percentage between species from different polyneopteran H_V_1 proteins.
**Fig. S1.** Amino acid sequences of polyneopteran insects proteins possessing a typical S4 RxWRxxR motif.
**Fig. S2.** Alignment of putative polyneopteran H_V_1 channels.
**Fig. S3.** Inside‐out patch‐clamp measurement of EtH_V_1.

## Data Availability

The experimental data generated and analysed during this study are included in this published article and are available from the corresponding author on reasonable request. The nucleotide sequence data that support the findings in this study are openly available in the European Nucleotide Archive (ENA) at EMBL‐EBI at https://www.ebi.ac.uk/ena/browser/view/ using the correspondent GenBank Accession Number shown in Table S1.
