# Supplementary material for: Voltage‐gated proton channels in polyneopteran insects
Source: FEBS Open Bio. 2022 Jan 19;12(2):523–37. doi: 10.1002/2211-5463.13361 (PMC8804609; doi:10.1002/2211-5463.13361)
Supplement: Supplementary file 1 — Table S1. List of all identified HV1 homologs compiled from TSA files and correspondent GenBank accession number. Table S2. Sequence identity percentage between species from different polyneopteran HV1 proteins. Fig. S1. Amino acid sequences of polyneopteran insects proteins possessing a typical S4 RxWRxxR motif. Fig. S2. Alignment of putative polyneopteran HV1 channels. Fig. S3. Inside‐out patch‐clamp measurement of EtHV1. [file FEB4-12-523-s001.docx]

**Supplementary data**

Table.S1. **List of all identified H_V_1 homologs compiled from TSA files and correspondent GenBank accession number.**

1. Polyneopteran H_V_1 proteins possessing a typical S4 RxWRxxR motif

| Species |  | GenBank Acc.No | length | SF/S1 |
| --- | --- | --- | --- | --- |
| Stenopelmatus sp. | Orthoptera; Ensifera | GDVX01019461 | 243 | D |
| Anostostoma australasiae | Orthoptera; Ensifera | GDYN01026133 |  |  |
| Phyllomimus sinicus | Orthoptera; Ensifera | GFRL01048039 |  | D |
| Clitarchus hookeri | Phasmatodea; Verophasmatodea | GFVY01085196 | 236 | E |
| **Extatosoma tiaratum** | **Phasmatodea; Verophasmatodea** | **GAWG01024136** | **236** | **E** |
| Cyphoderris sp. | Orthoptera; Ensifera; | GDWG01053486 |  | D |
| Sipyloidea sipylus | Phasmatodea; Verophasmatodea | GAWF01047109 | 237 | E |
| Entoria okinawaensis | Phasmatodea; Verophasmatodea | IADO01100848 | 238 | E |
| Ramulus artemis | Phasmatodea; Verophasmatodea | GAWE01048256 | 238 | E |
| Medauroidea extradentata | Phasmatodea; Verophasmatodea | GAWD01057497 | 243 | E |
| Orxines xiphias | Phasmatodea; Verophasmatodea | GDWL01008522 | 231 | E |
| Pseudophasma velutinum | Phasmatodea; Verophasmatodea | GDXC01031946 | 237 | E |
| Aretaon asperrimus | Phasmatodea; Verophasmatodea | GAWC01068486 | 230 | E |
| Hemiandrus sp. | Orthoptera; Ensifera | GDVH01038922 |  |  |
| Bacillus rossius | Phasmatodea; Verophasmatodea | GDTQ01031636 | 228 | E |
| Peruphasma schultei | Phasmatodea; Verophasmatodea | GAWJ02031075 | 236 | E |
| Carausius morosus | Phasmatodea; Verophasmatodea | GFAX01188248 |  | E |
| Eurycantha calcarata | Phasmatodea; Verophasmatodea | GDVD01008569 | 234 | E |
| Pseudosermyle phalangiphora | Phasmatodea; Verophasmatodea | GEAD01007790 |  | E |
| Ptilocerembia catherinae | Embioptera; Ptilocerembiidae | GDBY01020981 | 254 | D |
| Achrioptera fallax | Phasmatodea; Verophasmatodea | GDVF01001591 |  | E |
| Rhagadochir virgo | Embioptera; Scelembiidae | GDBX01026879 | 250 | D |
| Nippancistroger testaceus | Orthoptera; Ensifera | GCPE01026025 |  |  |
| Agamemnon cornutus | Phasmatodea; Verophasmatodea | GDCE01040333 |  |  |
| Tyrannophasma gladiator | Mantophasmatodea; Tyrannophasma | GDVU01017365 | 238 | E |
| Oreophoetes peruana | Phasmatodea; Verophasmatodea | GDWM01024205 |  | E |
| Karoophasma biedouwense | Mantophasmatodea; Austrophasmatidae; | GINP01204024 | 211 | E |
| Timema cristinae | Orthopteroidea; Phasmatodea | GAVX02021267 |  |  |
| Salmoperla sylvanica | Plecoptera; Perloidea | GIEJ01021403 |  |  |
| Trachyaretaon brueckneri | Phasmatodea; Verophasmatodea | GDBU01017397 |  |  |
| Tirachoidea westwoodii | Phasmatodea; Verophasmatodea | GCPH01037984 |  |  |
| Aposthonia japonica | Embioptera; Oligotomidae | GAWU01255994 | 273 | D |

1. Polyneopteran proteins with significant homology to other putative H_V_1 proteins without S4 coverage

| putative homologs without (complete) coverage of signature motiv |  | GenBank Acc.No. | length | S1 | BLAST identity/homology |
| --- | --- | --- | --- | --- | --- |
| Gampsocleis gratiosa | Orthoptera; Ensifera | GFSE01073908 |  |  | 31/49(63%), 42/49(85%) |
| Orthomeria sp. | Phasmatodea; Verophasmatodea | GCPP01011834 |  |  | 34/75(45%), 53/75(70%) |
| Megacrania phelaus | Phasmatodea; Verophasmatodea | GDWN01045762 |  | E | 61/73(84%), 66/73(90%) |
| Paranisacantha sp. | Phasmatodea; Verophasmatodea | GDYI01043373 |  |  | 51/75(68%), 62/75(82%) |
| Spathomorpha lancettifer | Phasmatodea; Verophasmatodea | GDYH01040357 |  |  | 48/77(62%), 57/77(74%) |
| Orestes mouhotii | Phasmatodea; Verophasmatodea | GDZN01037542 |  |  | 48/68(71%), 54/68(79%) |
| Abrosoma johorense | Phasmatodea; Verophasmatodea | GDWZ01005116 |  | E | 27/57(47%), 35/57(61%) |

1. Putative H_V_1 homologs found in insect TSA databases representing sample contamination

| likely sample contamination: |  |  | contamination | BLAST identity/homology to contamination |
| --- | --- | --- | --- | --- |
| Metallyticus splendidus | Mantodea; Metallyticidae | GATB01324360* | Fungi; Mucoromycota | 147/207(71%), 170/207(82%) |
| Forficula auricularia | Dermaptera | GAYQ01077212* | Fungi; Mucoromycota | 64/76(84%), 71/76(93%) |
| Gryllotalpa sp. | Orthoptera; Ensifera | GAWZ02038120 | Fungi; Dikarya | 79/99(80%), 86/99(86%) |
| Acanthocasuarina muellerianae | Hemiptera | GAYY01094200* | Fungi; Dikarya | 72/85(85%), 79/85(92%) |
| Nilaparvata lugens | Hemiptera | IACV01022379 | Fungi; Dikarya | 28/39(72%), 35/39(89%) |
| Ulopa reticulata | Hemiptera | GDEO01014017 | Fungi; Dikarya | 38/51(75%), 45/51(88%) |
| Aspidiotus destructor | Hemiptera | GGKE01114565 | Fungi; Dikarya | 35/53(66%), 41/53(77%) |
| Odontotaenius disjunctus | Holometabola | GDMS01028732 | Fungi; Mucoromycota | 204/206(99%), 205/206(99%) |
| Serangium japonicum | Holometabola | GGMU01156105 | Fungi; Dikarya | 236/237(99%), 236/237(99%) |
| Lepidotrigona ventralis | Holometabola | GFOP01018695 | Chelicerata; Arachnida | 114/229(50%), 156/229(68%) |

^* - samples were later removed.^

Table.S2. **Sequence identity percentage between species from different polyneopteran H_V_1 proteins.**

|  |  |  | St. sp. | Ph. si. | Ex. ti. | Eu. ca. | Pt. ca. | Rh. vi. | Ty. gl. | Ka. bi. |
| --- | --- | --- | --- | --- | --- | --- | --- | --- | --- | --- |
| Stenopelmatus sp. | Orthoptera | GDVX01019461 | 100 | **92** | 75 | 74 | 69 | 67 | 68 | 69 |
| Phyllomimus sinicus | Orthoptera | GFRL01048039 | **81** | 100 | 71 | 70 | 66 | 65 | 66 | 65 |
| Extatosoma tiaratum | Phasmatodea | GAWG01024136 | 54 | 49 | 100 | **91** | 68 | 66 | 66 | 69 |
| Eurycantha calcarata | Phasmatodea | GDVD01008569 | 55 | 52 | **83** | 100 | 70 | 68 | 65 | 67 |
| Ptilocerembia catherinae | Embioptera | GDBY01020981 | 54 | 55 | 45 | 51 | 100 | **95** | 65 | 65 |
| Rhagadochir virgo | Embioptera | GDBX01026879 | 52 | 53 | 44 | 47 | **91** | 100 | 66 | 63 |
| Tyrannophasma gladiator | Mantophasmatodea | GDVU01017365 | 42 | 43 | 42 | 42 | 44 | 46 | 100 | **90** |
| Karoophasma biedouwense | Mantophasmatodea | GINP01204024 | 46 | 44 | 47 | 48 | 45 | 46 | **82** | 100 |

Fig.S1. **Amino acid sequences of polyneopteran insects proteins possessing a typical S4 RxWRxxR motif.** Transmembrane segments S1-S4 are highlighted in yellow, and the putative selectivity filter in S1 in green. The three argenines and the tryptophan of the RxWRxxR motif are shown in blue and red, respectively.

Stenopelmatus sp. GDVX01019461

***M***ITHQGQGDHFYNEEEEYFRDEIDDNSEKSTSINKITSGHLPFREKARVVIHSQKFHIAVITLVIVDMFLVLFELLIDMSALQKDEHDWQHLLEFILKCCSISILSIFLLENIFKVYVMRAEFIHHYLEMFDAAVVVTSLILDIIFINEHNVTIGLGLIIILRLWRIVRVINGIVLTVKAQSDKLLEKEKQRRLRLLAKLKQIRQLCQVQHQEIKGLRCILDSHGIEIPTSAVLCHSITHPNN-

Anostostoma australasiae GDYN01026133

…HSWQHVLEFILKCCSISILSIFLLENVFKVYVMRAEFFHHYLEMFDAAVVVTSLILDIIFINEHNVTIGLGLIIILRLWRIVRVINGIILTVKAQSDKLLEKEKQRRLRLLAKLKQIRQLCQVQHQEIKGLRSILDSHGIEIPTSAVLCHSITHPNN-

Phyllomimus sinicus GFRL01048039

…IGVITLVIADVFLVVFELLIDMSALQKNQHEWQHLMEFILKCCSIVILSIFLLENIFKIYAMQIEFLQHYLEMFDAAVVVTSLIFDIVFINDHSTSIGLGLIIVLRLWRIVRVINGIVWTVKAQSDKLLEKEKRRHLRLLAKLKQIQQFCKIQHQEIKSLRHILDSHDIEIPKSPVFCHSISNVTK-

Clitarchus hookeri GFVY01085196

***M***GPNEAELETLSSDTSGPLRRYKQSRKPKGGFKRMETFREKAKFLLESPKFHIVVLTLVIIELVMVVVELMIDASGVEKSEALETAEIVLKYISISILSIFVIENLFTMYILRCEFFHKCLEVFDSIIVFTSLVLEVIFLNHHDAATGVGILIGLRLWRIVRVINGITVTVAAQSEKKLEEERNLREEVETELNKCVDRSKKQQCYILFLEKFIQSHGLEISPHSNCPSTNSSEGQ-

Extatosoma tiaratum GAWG01024136

***M***GPNEVELGTLSSDASGPLDRHKQSRKTQDGFERIKTFREKAKLLLESPKFHIVVLTLVIIELTMVVAELMIDASGVEKSEALETVEIALKFISISILSIFVIENLFTMYVLRCEFFHKCLEVFDSIVIFTSLVLEVIFLNHHDAATGVGILIGLRLWRIVRVINGIAVTVAAQSEKKLEEEKDLREEIEMELNKCVDRSKKQQCYILFLQKFIQSNGLEIPPHSSCPSRNSSEGH-

Cyphoderris sp. GDWG01053486

…RIIIHSQKFHIAVITLVIADVFLVLFELLIDMSALQKNEHDWHHLLEFVLKCCSISILSIFLLENIFKAYVMRVEFLHHYLEMFDAAVVVTSLIFDIIFINDHTTTTGLGLIIILRLWRIVRVINGIVLTVKAQSDKLLEKEKRRRLRLLAKLKQIRQLCRVQHQEIKGLKCILDSHGIEIPTSAVRCHSITNPND-

Sipyloidea sipylus GAWF01047109

***M***GPDDVELRTSLSGEASGRLYKHKQSRKPHNEVETKKPFREKARILLESPKFHIIVLVLVITELILVVVELMIDASGLKKNAQLEIVETIFKYISIFILSIFVVENLFTIYVLRCQFFHKCLEVFDIIVVVTSLVLEIVFFNHHDAATGVGILIGLRLWRIVRVVNGIVVTVTAQSEKKLEEEQELREEIESELKKCVIRSKKQQLYIQSLEKLLQSHGVVISTQLNGSARNSIDEK-

Entoria okinawaensis IADO01100848

***M***GQDEVELGSLNTGASGHLHSCDNSRKSRTGIERKKPFREKARLLLESPKFHIVVLALVIIELIMVVTELMIDASGVKKSEALETGEMALKYISIFILSVFVAENLFTVYVLRFEFFHKCLEVFDFIVVITSLVLEVIFLNHHDAATGVGILIGLRLWRIVRVVNGIAVTVTAQSEKKLEEERELREEMESKLNKCANVTKMQQNYIQNLEKLIKSHGLEIPRETSSPYRNSTDEQLT-

Ramulus artemis GAWE01048256

***M***GRDEVELGSLNTDTSGHLHIRNNSRKSRARIERKKSFREKARLLLESPRFHIVVLTLVILELTMVVAELMIDASGVEKSEALETGEIALKYISIFILSIFVAENLFTVYVLRCEFFHKCLEVFDFIVVITSLVLEVIFLNHHDAATGVGILIGLRLWRIVRVVNGIAVTVTAQSEKKLEEERELREEMESELNKCSNVTKTQQNYIQYLEKFIKSHGLEIPREPSNPYRNRTDEQLT-

Medauroidea extradentata GAWD01057497

***M***IGSVMGPDEVELGSLNTETKGHLHRNKKSRKSQHGFERKKPFREKARLLLESPKFHIVILTLVILELSMVVTELMIDASGVEKSEVLETVEIVLKYVSIFILSIFVAENLFTVYILRCEFFHKCLEVFDFIVVIMSLVLEVIFMNHHDAATGVGILIGLRLWRIVRVVNGIAVTVTAQSEKKLEEERELQEEIESELNKCVNVTKKQQHYILCLEKFIKSHGLEIPQEPSCLSKNSTDEQLT-

Orxines xiphias GDWL01008522

***M***SPDDVELQTSLGGDRHKHSREPHNGFERNKPFREKAKLLLESPKFHIVVLILVITELIMVVAELMIDASRVEKSEELELVEMILKYISIFILSIFVVENLFTMYVLRCQFFHKCLEMFDIVIVVTSLILEIIFLKHHDAATGVGILIGLRLWRIVRVINGIVVTVTAQSEKKLEEEKELREEIESELKKFISRTKKQQHYIQSLEKLLQSHGLEVPKQLSSTRNSIDEHM-

Pseudophasma velutinum GDXC01031946

***M***GPEEAELGTLSGDARATPRVHNQKRTTGTHGNFTRKKTFREKARLLLESPKFHIVVLTLVVAELIMVVTELMIDASGVEKSHTLETVEVVLKHIGILILSIFVAENLFTMYVLRCEFFGKCLEVFDTIVIVTSFVLEIVFLNHHDAASGFGILIALRLWRVVRVINGIVVTVTAQSEKKVEEERELREEAETELEKSHSLSEKQQQYIAHLEKFIQSHGLQVPPHPGQSPVHSTDQ-

Aretaon asperrimus GAWC01068486

***M***GPDDVELQTFNSDNSEHLHKRRQSRKSQDEVEKKKTFREKARLLLESPKFHIAVLTLVITELIMVVIELMIDASGVAKNEELETVEKILKCISIFILSLFIVENLFTLFVLRCEFFHNCLEVFDTIVVITSFVLEVVFLNRHDAATGVGILIGLRLWRIVRVINGIAVAVKAQLEKKLEEERELREETELELKKYIGLSKKQQRYILHLEKFIQTHGLEISPRNSVGDQ-

Hemiandrus sp. GDVH01038922

…ILSIFLLENIFKVYVMRDEFFHHYLEMFDAAVVVTSLILDIIFINEHNVTIGLGLIIILRLWRIVRVINGIILTVKAQSDKLLEKEKQRRLRLLAKLKQIRQLCQVQHQEIKGLRCILDSHGIEIPTSAVLCHSITHPNN-

Bacillus rossius GDTQ01031636

***M***GPDLEYAVISGDTSACPRASQQIKSSQHGLERKLPFREKARLLLESPKFHIAVLTLVVAELSMVVVELMIDASGVEKSWALEAVELTLKYASIAILSLFVLENLFTVYVLRCEFFRKCLEVFDTAVVVTSLVLEVYFLHRHDAANGVGILIALRLWRIVRVVNGIAVTVTAQSERKLDEERELREEAQSELRECVCARGKQQRYILQLEEFIRAHDLPVPPQPGGPP-

Peruphasma schultei GAWJ02031075

***M***GPEEAELGTLSGDASATPHVHNQKRFMGTHGNLSRKKTFREKARLLLESPKFHIVVLTLVVVELIMVVTELMIDASGVEKSDTLETVEVVLKHIGILILSIFVVENLFTMYVLRCEFFGKCLEVFDTIVIVTSFVLEIVFLNSHDAASGFGILIALRLWRVVRVINGIVVTVTAQSEKKVEEERELREEAETELVKSHSLSEKQRQYIAHLEKFIQSQGLQVPPHPGQSICSTDQ-

Carausius morosus GFAX01188248

…SCPETIYTESAARALCGHGTRTKQNTSVLKRKALTRREWINLWVNFVIVVLTLVIIELIMVVVELMIDASGVQKNETLEALEVALKYISISILSIFVAENFFTIYVLRCEFFHKCLEVFDTIVVVTSLVLEVIFLNHHDATTGIGILIGLRLWRIVRVVNGIAVTVTAQSEKKLEEERELREEIDSKLKECVNHSKKQQQYIMYLKKLLQSHGLEIPPQFSSSSRNSMVEE-

Eurycantha calcarata GDVD01008569

***M***GPDEAELQIFSNDAGRHLHSQSKKSPDEFEGKRTFREKARLLLESPKFHIVVLILVVLELTMVVVELMIDASGVEKNEVLKTVETVLKYVSIFILSIFVVENFFTIYVLRCEFFHKCLEVFDTIVVITSLVLEVIFFNHHDATTGIGILIGLRLWRIVRVINGIAVTVSAQSAKKLEEERELREEKESELKTCLGHYKKQQQYIMYLEKLLQSHGLEIPPQFSSSARNSSAEQ-

Pseudosermyle phalangiphora GEAD01007790

***M***GSDEVETLSCNTQTLHHRSKTTRRLQDEIEEKKTFREKSRLLLESPKFHIVVLTLVVTELAMVVIELMIDASGVEKSETLEMVEVVLKYLSIFILTIFVMESLFTLYVLRLEFFHKCLEVFDFIVVVTSLVLEVVFLNRHDAATGVGILIGLRLWRVVRV…

Ptilocerembia catherinae GDBY01020981

***M***TKIAETQDKEDQTTTVTLFEDEKLFQPELKRELSTSSESSDCSIRSQPTLRDKVYALHKSSEFHIAVLVLVVIDLLFVVTELAIDTDPTKSHDQNFILVENVLKYGSITILSLFLLENIFSIYVMRGEFFREYLEVFDAVVVVISLTVDIMVVSSHDTFSALGFIIVLRLWRIVRVITAVVTTVNDRAARKIKKEKEKRRNLQGELTEEIEIREKYEAELEFLRNLLEKSNSKNALSTPLSQLQTSNISRQKK-

Achrioptera fallax GDVF01001591

…NVIAVLTLVVAELTMVVVELMIDASGVEKSGPLEMLETVLKYISICILAIFVVENLFTLYVLRCEFFHKCLEVFDTVVVVTSLVLEVVFLNHHDAATGVGILIGLRLWRIVRVINGITVTVTAQSEKKFEEERELRIDMESRLKACASLSKKQQYYILQLEKFIQSHDLQIPPQLSHPSRDSIEEH-

Rhagadochir virgo GDBX01026879

***M***AKETNVTEKDQTTVKLFEDEKGRPQGLQRELSSTSEESDCSIKSSSSLKQKVHALHKSSEFHIAVLVLVVIDLLFVITELAIDTDPLKSHDKNFLIFENVLKYGSITILSLFLLENIFSIYVMGGDFFREYLEVFDFVVVVISLSADIMVVSSHDTFSALGFIIVLRLWRIVRVITAVVTTVNDRAARRIRKEKTKRRNLEAQLEEHVEIKEKYKSELEFLRNLLEKEQAKTTLTISPSQAKLAEMTRP-

Nippancistroger testaceus GCPE01026025

…YLEMFDAAVVVTSLILDINFISEHNVTIGLGLIIILRLWRIVRVINGIILTVKAQSDQLLEKETQRRLRLSAKLKQIRQLCQVQHQEIKGLRSILENHSIEIPTPAVLCHSINSNN-

Agamemnon cornutus GDCE01040333

…MVVVELMIDASDVEKSGTLETVELALKYFSISILSIFVVENMFTMYVLRCEFFHKCMEVFDTIVVVTSLVLEIIFLNHHDAATGVGILIGLRLWRIVRGINGIAVTVTAQSDKKVEEEREEREEIESRLKKCVDDSKKQQHYILQLEKFIHSQGLEIPVQLSCLRRDSIDEQ-

Tyrannophasma gladiator GDVU01017365

***M***RWLRCICTELMCCQRGQEADNRVTAAMRPGESSTSTTRLSEGDTFSSARGDGDHPRPSVDAQCSTWRDKCLPLLDSHHYHVSVVALTIVELLLGIGEILLDLSAMQQAGNKTLLDAERVLRLIGIVILHAFVLEIVFKICVLGTLFFRQPTELFDAVVIILSLVLDLYFLDHHDATIGLGFIIVLRLWRVVRLVTAVVTTVRMQTERKLQKEIRKRIEAEEKLEALLAEMERQKGVV-

Oreophoetes peruana GDWM01024205

…QIVVLALVVTELSMVVVELMIDASRVEKSEALETVELVLKYISISILSVFVAENLFTMYVLRCEFFHKCLEVFDTVVVVTSLVLEVVFLNRHDAATGVGILIGLRLWRIVRVVNGIAVTVSAQSEKKLEEERELREEAQAGLKKTSALCERQQQYIQRLEEFILSHGLSVPPQPTTSRLGPNIRAMHVSGI-

Karoophasma biedouwense GINP01204024

***M***WPIEEADMMGLPKDDTLSIRTDDGDCSGLSEDPQPSTWRGTCRVLLESRQYHVSVVALTLIELLLVIAELLLDMSAMQHMGDKSLLDTERVLRLIGIAILHIFVLEIVFKICVLRTHFFRHPTELFDAVVVILSLVLDLCFLDHHDATVGLGFIIVLRLWRIVRVVTGVVTTVRLEMERKLQKEIKKRIEVEKRLDTLLAEMEQLKGVVS-

Timema cristinae GAVX02021267

…TILSIFVVENLFTIYVMGWDFFKHFLEVFDFTIVLTSLVLDIVFMHSHAQAGFGLLIILRLWRIVRVINGIVVAMSSQA…

Salmoperla sylvanica GIEJ01021403

…FKSKMEIFDAFIIITSLALDIVFFQHNDAALLIGDLIIVLRLWRIVRVIHGIVLSVSTPIEHNLEKE…

Trachyaretaon brueckneri GDBU01017397

…FVLEVVFLNRHDAATGVGILIGLRLWRIVRAINGIAVAVKAQLEKKLEEERELREETEAELKKYIGLSKKQQRYILHLEKFIQTHGLEISPRNSIDDQ-

Tirachoidea westwoodii GCPH01037984

…VFLNRHDAATGVGILIGLRLWRIVRVINGIAVTVTVQSEKKSEEEREIREEMESALNKCVDSNKKQQHYILCLEKFIQSHGLQIPPQPSCPSRNSSDEQ-

Aposthonia japonica GAWU01255994

***M***AKGTYTVDNDHQTIVNLLGDEKVPSPEFVRELSTDSSDCSIKSQTTLRDKVHALHKSSEFHIAVLVLVVIDLLFVITELAIDTDPIKSHDPSFIIFENILKYGSITILSVFLLENIFSIYVMRGDFFREYLEVFDAVVVVISLTVDIMVVSSHDTFSALGFVIVLRLWRIIRVITAVVTTVNDRAERRIRKQKERRRRAEEDLAEHKEIKEKHEAELEFLRNLVHKNNYKNTLTPQVHETRRSGRYIHNIISSTQSIMSNIMNSTHSIASHK-

Fig.S2. **Alignment of putative polyneopteran H_V_1 channels.** As a comparisson, human H_V_1 (hH_V_1) and sequences of four basal hexapodes, *Catajapyx aquilonaris* (CaH_V_1, Diplura), *Filientomon takanawanum* (FtH_V_1,Protura), *Pedetontus okajimae* (PoH_V_1, Archaeognatha), *Nicoletia phytophila* (NpH_V_1, Zygentoma), were aligned with seven polyneopteran sequences from *Extatosoma tiaratum* (EtH_V_1, Phasmatodea), *Sipyloidea sipylus* (SsH_V_1, Phasmatodea), *Ramulus artemis* (RaH_V_1, Phasmatodea), *Medauroidea extradentata* (MeH_V_1, Phasmatodea), *Aretaon asperrimus* (AaH_V_1, Phasmatodea), *Peruphasma schultei* (PsH_V_1, Phasmatodea) and *Aposthonia japonica* (AjH_V_1, Embioptera). In addition, the *Metallyticus splendidus* (MsH_V_1) sequence representing a fungal contamination was added in the bottom line of the alignment, the fungal typical exchange arginine R3 to lysine within the voltage-sensor position is indicated by an arrow. Transmembrane regions are shown in yellow, the selectivity filter shown in light green (D) or dark green (E), the voltage-sensor residues in S4 are indicated in blue and the H_V_1-typical tryptophan residue in S4 in marked in red.

**Polyneopteran and basal hexapod Hv1 Alignment**

....|....| ....|....| ....|....| ....|....| ....|....|

10 20 30 40 50

hH_V_1 MATWDEKAVT RRAKVAPAER MSKFLR---- --------HF TVVGDDYHAW

CaH_V_1 --------MQ RHRRLSEENV EGVVVAEAAV DMGGEGSKHQ QGMRNNKKDM

FtH_V_1 --------MG SEIELIDYQ- ---------- ---------- ----------

PoH_V_1 --------ML GHRRLSEDAA S--------- ---------- ----------

NpH_V_1 --------MW LKMDAHKRLS ---------- ---------- ----------

EtH_V_1 ---------M GPNEVELGT- ---------- ---------- ----------

SsH_V_1 ---------M GPDDVELRTS ---------- ---------- ----------

RaH_V_1 ---------M GRDEVELGS- ---------- ---------- ----------

MeH_V_1 ----MIGSVM GPDEVELGS- ---------- ---------- ----------

AaH_V_1 ---------M GPDDVELQT- ---------- ---------- ----------

PsH_V_1 ---------M GPEEAELGT- ---------- ---------- ----------

AjH_V_1 -------MAK GTYTVDNDHQ TIVNL----- ---------- ----------

MsH_V_1-fungal --------MS ASYGSLPTH- ---------- ---------- ----------

....|....| ....|....| ....|....| ....|....| ....|....|

60 70 80 90 100

hH_V_1 NINYKKWENE EEEEEEEQPP PTPVSGEEGR AAAPDVAPAP GPAPRAPLDF

CaH_V_1 ATSRRRTAAE EEDEDEDD-- ---DSCESDM EGSSVEVGPV RDDTIKSMGF

FtH_V_1 -QAQPRTFDE RLS------- ---------- KECYISVDDS DVYYRH-VTF

PoH_V_1 AVSDRSALNQ HESHE----- ---------- ---------- -------MTF

NpH_V_1 -EDLEKVIMK EDG------- ---------- NSSIMTEPDH NIQPS--KTV

EtH_V_1 -LSSDASGPL DRH------- ---------K QSR--KTQDG FERI---KTF

SsH_V_1 -LSGEASGRL YKH------- ---------K QSR--KPHNE VETK---KPF

RaH_V_1 -LNTDTSGHL HIR------- ---------N NSR--KSRAR IERK---KSF

MeH_V_1 -LNTETKGHL HRN------- ---------K KSR--KSQHG FERK---KPF

AaH_V_1 -FNSDNSEHL HKR------- ---------R QSR--KSQDE VEKK---KTF

PsH_V_1 -LSGDASATP HVH------- ---------N QKRFMGTHGN LSRK---KTF

AjH_V_1 -LGDEKVPSP EFV------- ---------R ELSTDSSDCS IKSQ---TTL

MsH_V_1-fungal ---DPEEAQE DQ-------- ---------- -----VTMEE EKRG-----W

....|....| ....|....| ....|....| ....|....| ....|....|

110 120 **S1** 130 140 150

hH_V_1 RGMLRKLFSS HRFQVIIICL VVLDALLVLA ELILDLKI-- ----------

CaH_V_1 RGKAKAVLEA HRFHLAVLFL VLLDCLMVIG QILLDLER-- ----------

FtH_V_1 RSWVRILLHA HKYQIFVILL VLFDSFLVIV ELILEMEFH- ----------

PoH_V_1 RQKTVILLES HKFQIGVISL VILDCIIVIV ELILDIEAAV ----------

NpH_V_1 RERLRKLLHS HKFQISVITL VIIDCLLVIT ELLIDLEM-- ----------

EtH_V_1 REKAKLLLES PKFHIVVLTL VIIELTMVVA ELMIDASG-- ----------

SsH_V_1 REKARILLES PKFHIIVLVL VITELILVVV ELMIDASG-- ----------

RaH_V_1 REKARLLLES PRFHIVVLTL VILELTMVVA ELMIDASG-- ----------

MeH_V_1 REKARLLLES PKFHIVILTL VILELSMVVT ELMIDASG-- ----------

AaH_V_1 REKARLLLES PKFHIAVLTL VITELIMVVI ELMIDASG-- ----------

PsH_V_1 REKARLLLES PKFHIVVLTL VVVELIMVVT ELMIDASG-- ----------

AjH_V_1 RDKVHALHKS SEFHIAVLVL VVIDLLFVIT ELAIDTDP-- ----------

MsH_V_1-fungal RHSLGERLES QNFHMTVLAL VLIDATCVAI QIIYTFFHEC QVPVITSTMV

....|....| ....|....| ....|....| ....|....| ....|....|

160 170 **S2** 180 190 200

hH_V_1 --IQPDKNNY AAMVFHYMSI TILVFFMMEI IFKLFVF--- --RLEFFHHK

CaH_V_1 ----VDHNHP SQQALHWASL IILCVFTLEF WLKVYVYHM- -ELFRDKRHR

FtH_V_1 -----LADTL APKCLHFLSI SILILFIVEI FFKIYAM--- --GLVYFISF

PoH_V_1 NKENAHLYHD ALSVLHYTSL SILCIFLVEI GMKVYAF--- --RFGFCSLH

NpH_V_1 ----HEEESL AQHVLHYCSI TILSIFIVEI FLKLYAF--- --RQEFFKHR

EtH_V_1 VEKSE-ALET VEIALKFISI SILSIFVIEN LFTMYVL--- --RCEFFHKC

SsH_V_1 LKKNA-QLEI VETIFKYISI FILSIFVVEN LFTIYVL--- --RCQFFHKC

RaH_V_1 VEKSE-ALET GEIALKYISI FILSIFVAEN LFTVYVL--- --RCEFFHKC

MeH_V_1 VEKSE-VLET VEIVLKYVSI FILSIFVAEN LFTVYIL--- --RCEFFHKC

AaH_V_1 VAKNE-ELET VEKILKCISI FILSLFIVEN LFTLFVL--- --RCEFFHNC

PsH_V_1 VEKSD-TLET VEVVLKHIGI LILSIFVVEN LFTMYVL--- --RCEFFGKC

AjH_V_1 IKSHDPSFII FENILKYGSI TILSVFLLEN IFSIYVM--- --RGDFFREY

MsH_V_1-fungal HPSKESFLFI AFELAEVFSI TICFLFMIEI ILSLIAFGPK YFLPGWPHWK

....|....| ....|....| ....|....| ....|....| ....|....|

**S3** 210 220 230 240 **S4** 250

hH_V_1 FEILDAVVVV VSFILDIVLL ----FQEHQF EALGLLILLR LWRVARIING

CaH_V_1 MELFDGAIVM ISLALDIIFY ----KHHGAA SALGLLVVLR FWRVARILNA

FtH_V_1 WEVFDGSIVI LSFAFDVVFR DSHSNSHHHA NGSGLLIVLR LWRVVRVVNG

PoH_V_1 LEVFDALIII VSFIIYIVFR ----NSHDAS DGLRFVIILR LWRIARIVNS

NpH_V_1 LEVFDAIIVI VSFALDIAFR ----NSRDAL SGVGLIIILR LWRVARVLNG

EtH_V_1 LEVFDSIVIF TSLVLEVIFL ----NHHDAA TGVGILIGLR LWRIVRVING

SsH_V_1 LEVFDIIVVV TSLVLEIVFF ----NHHDAA TGVGILIGLR LWRIVRVVNG

RaH_V_1 LEVFDFIVVI TSLVLEVIFL ----NHHDAA TGVGILIGLR LWRIVRVVNG

MeH_V_1 LEVFDFIVVI MSLVLEVIFM ----NHHDAA TGVGILIGLR LWRIVRVVNG

AaH_V_1 LEVFDTIVVI TSFVLEVVFL ----NRHDAA TGVGILIGLR LWRIVRVING

PsH_V_1 LEVFDTIVIV TSFVLEIVFL ----NSHDAA SGFGILIALR LWRVVRVING

AjH_V_1 LEVFDAVVVV ISLTVDIMVV ----SSHDTF SALGFVIVLR LWRIIRVITA

MsH_V_1-fungal LHVFDAAVIS TTFVLEVGLR ----GKEREI A--GLLIVFR LWRVVKVVEA

↑

....|....| ....|....| ....|....| ....|....| ....|....|

260 270 280 290 300

hH_V_1 IIISVKTRSE RQLLRLKQMN VQLAAKIQHL EFSCSEKEQE IERLNKLLRQ

CaH_V_1 AVMAVQTTAQ SQLEIERKKY ATLRLKYT-- EVHEELR--- --NLKLVLEM

FtH_V_1 VVLSVRAQAN FRLHKEKVAK QLVEAELKVS QAQCLFYQKQ IRTMCQLLDE

PoH_V_1 IIVPVKKAAE KKFEDEHFRR MRLEEEVARL R--------- -EIIR----Q

NpH_V_1 VVLSVKMQAE HQLEREKQRG MALEGELSRC RQVCAAQQRE LDVLRAVLQH

EtH_V_1 IAVTVAAQSE KKLEEEKDLR EEIEMELNKC VDRSKKQQCY ILFLQKFIQS

SsH_V_1 IVVTVTAQSE KKLEEEQELR EEIESELKKC VIRSKKQQLY IQSLEKLLQS

RaH_V_1 IAVTVTAQSE KKLEEERELR EEMESELNKC SNVTKTQQNY IQYLEKFIKS

MeH_V_1 IAVTVTAQSE KKLEEERELQ EEIESELNKC VNVTKKQQHY ILCLEKFIKS

AaH_V_1 IAVAVKAQLE KKLEEERELR EETELELKKY IGLSKKQQRY ILHLEKFIQT

PsH_V_1 IVVTVTAQSE KKVEEERELR EEAETELVKS HSLSEKQRQY IAHLEKFIQS

AjH_V_1 VVTTVNDRAE RRIRKQKERR RRAEEDLAEH KEIKEKHEAE LEFLRNLVHK

MsH_V_1-fungal VVMSVSFAHQ EELDQLKVSY AELETKLELE EEKNKQL--- ----------

....|....| ....|....| ....|....| ....|....| ....|.

310 320 330 340

hH_V_1 H--------- ---------- --------GL LGEVN----- ------

CaH_V_1 SGIVLPSTN- ---------- ----NQGNHN PQEAAPE-QS SGVTSE

FtH_V_1 RSIPYQVAHF VP-------- ----GKVVNE LPQPHCTT-- TSMY--

PoH_V_1 HGIAVDLE-- ---------- ---------- ---------P DRI---

NpH_V_1 HGLDQQLPDG NR-------- ----VDVVAD VEKR------ ------

EtH_V_1 NGLEIPPHSS CP-------- ------SRNS SEGH------ ------

SsH_V_1 HGVVISTQLN GS-------- ------ARNS IDEK------ ------

RaH_V_1 HGLEIPREPS NP-------- ------YRNR TDEQLT---- ------

MeH_V_1 HGLEIPQEPS CL-------- ------SKNS TDEQLT---- ------

AaH_V_1 HGLEISP--- ---------- ------RNSV GDQ------- -----.

PsH_V_1 QGLQVPPHP- GQ-------- ------SICS TDQ------- ------

AjH_V_1 NNYKNTLTPQ VHETRRSGRY IHNIISSTQS IMSNIMNSTH SIASHK

Fig.S3. **Inside-out patch-clamp measurement of EtH_V_1. A)** Internal pH_i_ is exchanged from 6.5 to 7.0 in an inside-out patch-clamp configuration. Families of pulses were applied in 10 mV increments from the holding potential (- 70 mV for pH_i_ = 6.5 and -30 mV for pH_i_ = 7.0) to the shown potential. *V*_rev_ was ~ 0 mV and ~ 35 mV for pH_i_ = 6.5 and pH_i_ = 7.0, respectively. pH conditions are depicted as pH_o_ // pH_i_. **B)** Conductance-voltage plot of measurements in A, showing a *g*_H_*V* rightward shift of ~ 22 mV. C) Activation kinetics plot of recordings from A. In symmetrical pH_o_ // pH_i_, the voltage-dependence of τ_act_ is ~ - 0.1 *e*-fold s ∙ mV^-1^.
